# Robust and specific ratiometric biosensing using a copper-free clicked quantum dot–DNA aptamer sensor†
†Electronic supplementary information (ESI) available: Details on the synthesis, purification and characterisation of the DHLA–PEG600–N_3_, cyclooctyne–DNA, and QD–TBA_20_ conjugates as well as all supporting figures and tables. See DOI: 10.1039/c3nr02897fClick here for additional data file.



**DOI:** 10.1039/c3nr02897f

**Published:** 2013-09-11

**Authors:** Haiyan Zhang, Guoqiang Feng, Yuan Guo, Dejian Zhou

**Affiliations:** a School of Chemistry and Astbury Centre for Structural Molecular Biology , University of Leeds , Leeds LS2 9JT , UK . Email: y.guo@leeds.ac.uk ; Email: d.zhou@leeds.ac.uk; b Key Laboratory of Pesticide and Chemical Biology of Ministry of Education , College of Chemistry , Central China Normal University , 152 Luoyu Road , Wuhan 430079 , P.R. China

## Abstract

We report herein the successful preparation of a compact and functional CdSe–ZnS core–shell quantum dot (QD)–DNA conjugate *via* highly efficient copper-free “click chemistry” (CFCC) between a dihydro-lipoic acid–polyethylene glycol–azide (DHLA–PEG–N_3_) capped QD and a cyclooctyne modified DNA. This represents an excellent balance between the requirements of high sensitivity, robustness and specificity for the QD-FRET (Förster resonance energy transfer) based sensor as confirmed by a detailed FRET analysis on the QD–DNA conjugate, yielding a relatively short donor–acceptor distance of ∼5.8 nm. We show that this CFCC clicked QD–DNA conjugate is not only able to retain the native fluorescence quantum yield (QY) of the parent DHLA–PEG–N_3_ capped QD, but also well-suited for robust and specific biosensing; it can directly quantitate, at the pM level, both labelled and unlabelled complementary DNA probes with a good SNP (single-nucleotide polymorphism) discrimination ability in complex media, *e.g.* 10% human serum *via* target-binding induced FRET changes between the QD donor and the dye acceptor. Furthermore, this sensor has also been successfully exploited for the detection, at the pM level, of a specific protein target (thrombin) *via* the encoded anti-thrombin aptamer sequence in the QD–DNA conjugate.

## Abstract 2

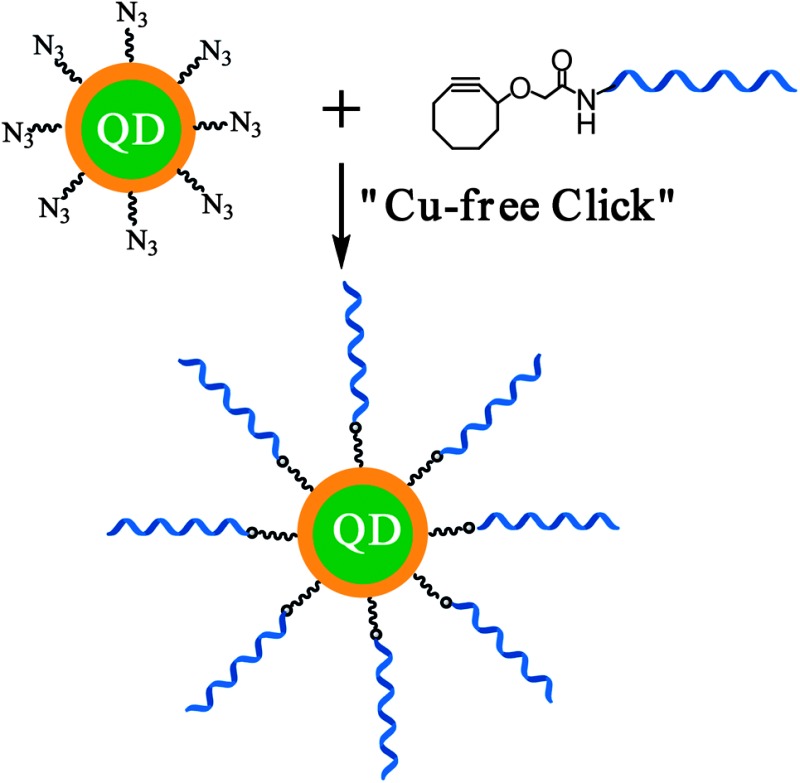
A robust and copper-free clicked quantum dot–DNA sensor for sensitive and ratiometric detection of specific DNA and protein targets at the pM level is reported.

## Introduction

The unique, size-dependent, highly stable and bright fluorescence of quantum dots (QDs) make them powerful tools in broad ranges of bio-related applications.^1–4^ In particular, their broad absorption and narrow and symmetric emission are extremely well-suited for Förster resonance energy transfer (FRET) based sensing, because these spectral characteristics enable a wide selection of excitation wavelengths to minimise direct excitation of the acceptor, reducing the background and hence improving the sensitivity.^1,2^ Indeed, numerous QD-FRET based biosensors have been reported.^3,4^ Despite these, the sensitivity and specificity of the QD-FRET based biosensors have largely been limited by challenges in preparing compact and functional QD-bioconjugates that are stable and effectively resist non-specific adsorption.^2–4^ For example, water-soluble QDs prepared by ligand exchange are compact, but they often show low stability in biologically relevant buffers and resistance to non-specific adsorption, limiting their sensing specificity and robustness.^2^ Whereas those prepared by physical encapsulation with amphiphilic polymers and/or PEGylated lipids (on which most commercial water-soluble QDs are based) are stable and can resist non-specific adsorption, but their large size (with hydrodynamic radii often greater than the *R*_0_ of most QD–dye FRET pairs even prior to bioconjugation)^2^ can greatly limit their FRET efficiency (sensitivity). Although the FRET efficiency can be enhanced by increasing the ratio of acceptors on each QD, such designs are inefficient at low target to QD ratios.^2^ Therefore for biosensing, it is important to balance the requirements of sensitivity and robustness because they are often incompatible. In this regard, QDs capped with PEGylated small-molecule ligands appeared to be highly attractive; they are relatively compact yet display good stability and, more importantly, effective resistance to non-specific adsorption of biomolecules.^2^

Besides surface capping, a robust and efficient QD-bioconjugation chemistry that can offer high bioactivity without compromising the QD fluorescence is also important. In this regard, the Cu(i) catalysed azide–acetylene cycloaddition, best known as the “click chemistry” (CuCC),^5*a*^ is highly powerful and versatile; it offers exquisite functional group selectivity and high yield. It has been used successfully in preparing a wide range of functional nanoparticle bioconjugates (*e.g.* gold, magnetic, silica and polymer nanoparticles) for sensing and biomedical applications.^5^ However, the CuCC is unsuitable for the QD, because the Cu(i) catalyst used in the CuCC can efficiently and irreversibly quench the QD fluorescence.^6^ The Cu-free “click chemistry” (CFCC) between strained cyclooctynes and azides happens rapidly and efficiently, and moreover, it does not require any Cu catalyst.^7^ Therefore, the CFCC appears to be ideal for efficient QD-bioconjugation without compromising the QD fluorescence.^7*a*^ Indeed, the CFCC has been successfully used to make functional QD–protein/small-molecule conjugates recently for live virus labelling/imaging and intra-cellular trafficking studies.^8^ Despite such developments, the QDs used in these studies were all capped with polymer based ligands (and hence of relatively big sizes) because the QD sizes here are less critical for such applications.^8^ To our knowledge, the CFCC has yet to be used to develop QD-FRET based biosensors where the compact size of the QD-bioconjugate is known to be of critical importance. Herein, we report the successful preparation of the first compact and functional QD–DNA conjugate *via* the CFCC between a dihydrolipoic acid–polyethylene glycol–azide (DHLA–PEG–N_3_) capped CdSe–ZnS core–shell QD and a cyclooctyne modified DNA, giving a good balance between the requirements of high sensitivity, specificity and robustness. This is supported by a FRET analysis showing a relatively short QD-dye distance of ∼5.8 nm for the QD–DNA FRET system. Moreover, the CFCC clicked QD–DNA conjugate is found not only to retain the native fluorescence quantum yield (QY) of the parent QD, but also well-suited for robust biosensing; it can directly quantitate, at the pM level, both labelled and unlabelled complementary DNA probes with a good SNP (single-nucleotide polymorphism) discrimination ability even in complex media, *e.g.* 10% human serum, on a conventional fluorimeter. It can also directly detect, at the pM level, a specific protein *via* the encoded DNA aptamer sequence.

## Results and discussion

### CFCC based QD–DNA conjugation and sensing principle

**Scheme 1:**
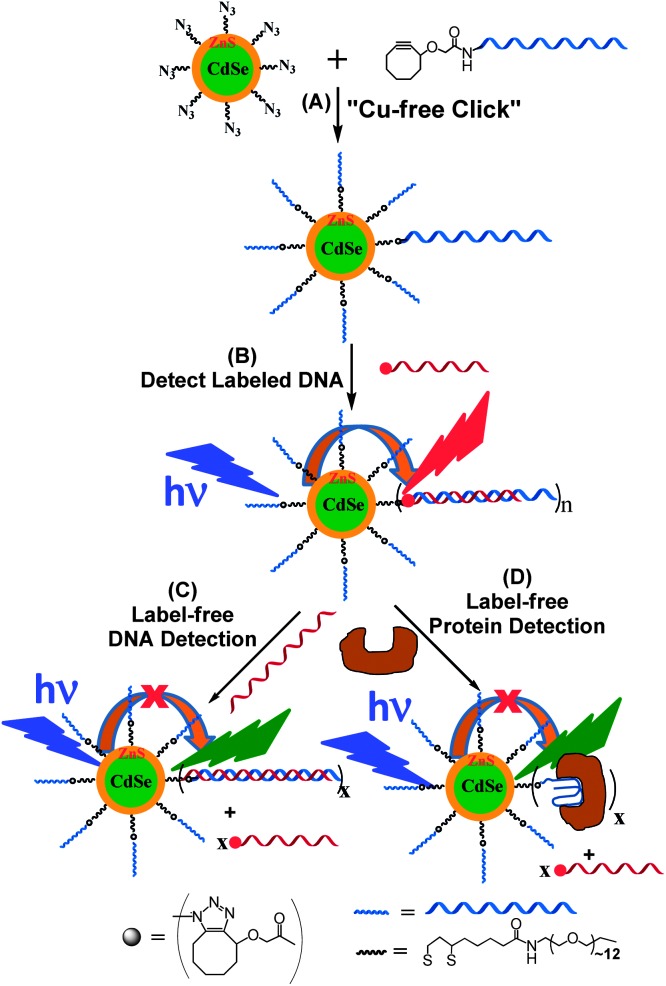
(A) Schematic approach to the Cu-free “clicked” QD–DNA conjugate. (B) Hybridization of a complementary dye-labelled DNA probe with the QD–DNA conjugate leads to QD sensitized dye FRET signals as a readout for labelled DNA detection. (C) Incubation of the QD–double-stranded (ds) DNA conjugate formed in (B) with a longer, unlabeled DNA displaces the shorter labelled DNA reporter, reducing the QD to dye FRET for label-free DNA detection. (D) Incubation of the QD–dsDNA conjugate (B) with a target protein that binds to the encoded aptamer sequence in the QD–dsDNA conjugate displaces the reporter DNA, leading to reduction of QD to dye FRET for label-free protein detection. The block arrows give the FRET directions.

Scheme 1 shows our approach to the QD–DNA conjugate *via* the CFCC and its use in label- and label-free-detection of DNA and protein targets *via* target binding induced changes in the QD sensitized dye FRET signals. First, a multi-functional ligand, containing a dihydrolipoic acid (DHLA, for strong QD binding) head group, a polyethylene glycol moiety of a molecular weight of 600 (PEG600, for providing good water-solubility and effective resistance to non-specific adsorption of biomolecules) and a terminal azide group (for efficient DNA conjugation *via* the CFCC), DHLA–PEG600–N_3_, was prepared (see the ESI† for details).^9,10^ A PEGylated DHLA ligand was used as the QD surface capping ligand here because it represented an excellent balance between the requirements of high stability and resistance to non-specific adsorption (for robust biosensing) and the structural compactness (for high sensitivity).^2^ Then a hydrophobic CdSe–ZnS core–shell QD (*λ*_EM_ ∼ 605 nm, QY ∼ 20%, capped with hydrophobic trioctyl-phosphine oxide/trioctylphosphine) was made water-soluble by ligand exchange with the DHLA–PEG600–N_3_ in a mixed solvent of CHCl_3_–ethanol using our previously established procedures,^3*l*^ yielding the QD–DHLA–PEG600–N_3_ which was readily soluble in polar solvents. Its fluorescence QY was found to decrease to ∼6.0% (and hence a decrease of *ca.* 70%), which is in good agreement with most other reports in the literature where most hydrophobic CdSe–ZnS core–shell QDs typically showed a QY decrease of 50–80% following the ligand exchange and transfer to aqueous media.^3,4^ A single-stranded (ss) target DNA encoded with a 29 mer anti-thrombin (TB) aptamer sequence with strong affinity for TB (*K*_d_ ∼ 0.5 nM, modified with a C_6_-amine at 5′, H_2_N–TBA, see Table 1)^11^ was reacted with an *N*-hydroxysuccinimide (NHS) ester activated cyclooctyne to yield TBA–cyclooctyne, which was then reacted with the QD–DHLA–PEG600–N_3_ in a mixed solvent of ethanol–water at a molar ratio of 30 : 1. This led to QD–TBA covalent conjugation *via* the efficient CFCC approach. Approximately 20 strands of TBAs were found to be conjugated to each QD, denoted as QD–TBA_20_ hereafter, this gave a DNA conjugation efficiency of ∼67%. The detailed experimental procedures for the ligand synthesis and QD–DNA conjugation are given in the ESI.† The QY of the resulting QD–TBA_20_ was determined as ∼5.9% using rhodamine 6G in ethanol as the calibration standard (QY 95%),^3*b*^ which is effectively the same as that of the QD–DHLA–PEG600–N_3_ (*ca.* 6.0%).

**Table 1:** The DNA sequences and their abbreviations used in this paper. TBA is modified with C_6_NH_2_ at 5′, and all other DNAs are labelled with an Atto-647N at 3′. The sequences of DNA29, DNA18, DNA15 and DNA12 are fully complementary to TBA, but DNA12-SM contains a single-base mismatch (shown in bold italic). The 29 mer anti-thrombin aptamer sequence encoded in TBA is shown in italic. DNA29-NL has the same sequence as DNA29 but without the Atto647N label

DNA code	Sequence
TBA	5′-TT*AGTCCGTGGTAGGGCAGGTTGGGGTGACT*-3′
DNA29	3′-TCAGGCACCATCCCGTCCAACCCCACTGA-5′
DNA18	3′-AATCAGGCACCATCCCGT-5′
DNA15	3′-AATCAGGCACCATCC-5′
DNA12	3′-TCAGGCACCATC-5′
DNA12-SM	3′-TCAG***A***CACCATC-5′
DNA-NC	3′-TAGTCC CGATT TCTCACG-5′

The QD–TBA_20_ was found to be highly soluble and stable in aqueous media. It showed no change of physical appearance or fluorescence after being stored in a fridge at 4 °C for over two months. More importantly, the QD–TBA_20_ was found to have effectively retained the native QY of the parent QD–DHLA–PEG600-N_3_. In contrast, conjugation of the H_2_N–TBA to a water-soluble, glutathione capped QD (the same batch of QD) by using 1-ethyl-3-[3-dimethylaminopropyl] carbodiimide (EDC)–NHS mediated covalent coupling resulted in significantly reduced QY. The fluorescence intensity of the former was ∼6.4 times as strong as the latter (see ESI, Fig. S1†) despite that the latter exhibited a higher QY in pure water (∼18%). Moreover, the number of TBA strands conjugated to each QD by the CFCC (20) was also 4 times that of the latter (∼5). All these demonstrate that the CFCC based QD–DNA conjugation approach developed herein is far superior over the EDC–NHS mediated coupling, a conventional widely used QD-bioconjugation method,^3^ in terms of both the DNA conjugation efficiency and ability of maintaining a high QY of the QD.

### FRET analysis of the CFCC clicked QD–DNA conjugate

Prior to using the CFCC clicked QD–DNA conjugate for sensing, a FRET analysis on the CFCC clicked QD–TBA_20_ after hybridisation with a complementary strand (DNA29) of different molar ratios was carried out to ensure a relatively small donor–acceptor distance (*r*) for high sensitivity. This is because the FRET efficiency (*E*) decreases dramatically with the increasing *r* value following the Förster dipole–dipole interaction formula:

1 *E* = 1/[1 + (*r*/*R*_0_)^6^]

where *R*_0_ is the Förster radius of the single donor (QD)–single acceptor (Atto647N) FRET pair here, for which *E* = 50%. *R*_0_ can be estimated from the spectral overlap (*I*) and the QY of the QD donor *via* the following equation:2
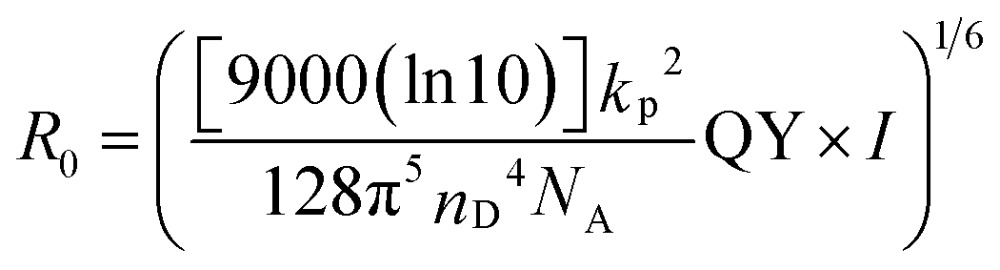
where *n*_D_ is the refractive index of the medium (estimated as 1.4 here), *N*_A_ is Avogadro's number (6.02 × 10^23^), *k*_p_^2^ is the orientation factor (2/3 here assuming randomly orientated fluorophores). The integral of the spectral overlap, *I*, is defined as:3
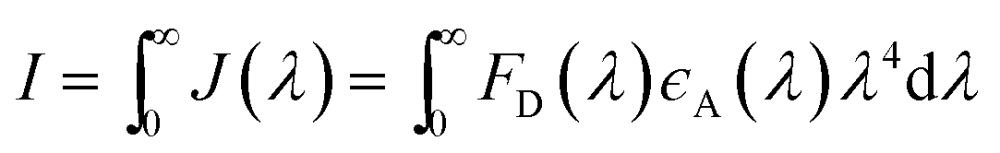



*I* is the integral of the donor–acceptor spectral overlap over all wavelengths *λ*, *F*_D_(*λ*) is the normalized donor emission and *ε*_A_ is the absorption extinction coefficient of the acceptor.

The normalised absorption and emission spectra of the QD and Atto647N and the corresponding spectral overlap function are shown in the ESI, Fig. S2.† A significant overlap between the QD (donor) emission and Atto647N (acceptor) absorption is clear, suggesting that they can have efficient FRET. The spectral overlap, *I*, can be calculated from Fig. S2B,† giving a value of 6.68 × 10^15^ M^–1^ cm^–1^ nm^4^. These combined with the QY of the QD (5.9%) and the parameters above yielded a *R*_0_ value of 4.25 nm for the QD–Atto647N FRET pair (at 1 : 1 molar ratio).

For a single-donor (QD here) simultaneously FRET with *n* identical acceptor systems, *E* is given by the following equation:

4 *E* = *nR*_0_^6^/[*r*^6^ + *nR*_0_^6^]

where the apparent *E* can be estimated directly from the acceptor fluorescence enhancement *via* the following equation:

5 Apparent *E* = *I*_A_/[*I*_A_ + *I*_D_]

where *I*_A_ and *I*_D_ are the integrated acceptor and donor fluorescence, respectively. Here a ratiometric FRET analysis is used which can be more reliable than those only based on donor quenching because it can be essentially insensitive to instrument noise and signal fluctuations, making the analysed result potentially more accurate. Moreover, *I*_A_ here only comes from the QD-sensitised FRET because the acceptor is not directly excited under our experimental conditions (see the next section below) and any unbound species will be too far away to participate in the FRET process, and hence do not interfer with target detection, allowing highly convenient and separation-free measurements.

The FRET study was carried out with 2 nM of the QD–TBA_20_ sample after hybridization with different molar equivalents of DNA29 (3′-labelled with an Atto647N acceptor, see Table 1). Hybridization of DNA29 with the QD–TBA_20_ should bring the Atto647N acceptor in the close proximity to the QD, leading to the QD sensitized Atto-647N FRET signal upon excitation of the QD (Scheme 1B). For eqn (4) to be valid, all DNA29 strands introduced (and Atto647N labels) should bind to the QD–TBA_20_. Hence the longest DNA29 probe which forms the most stable duplex with the QD–TBA_20_ was used.

**Fig. 1:**
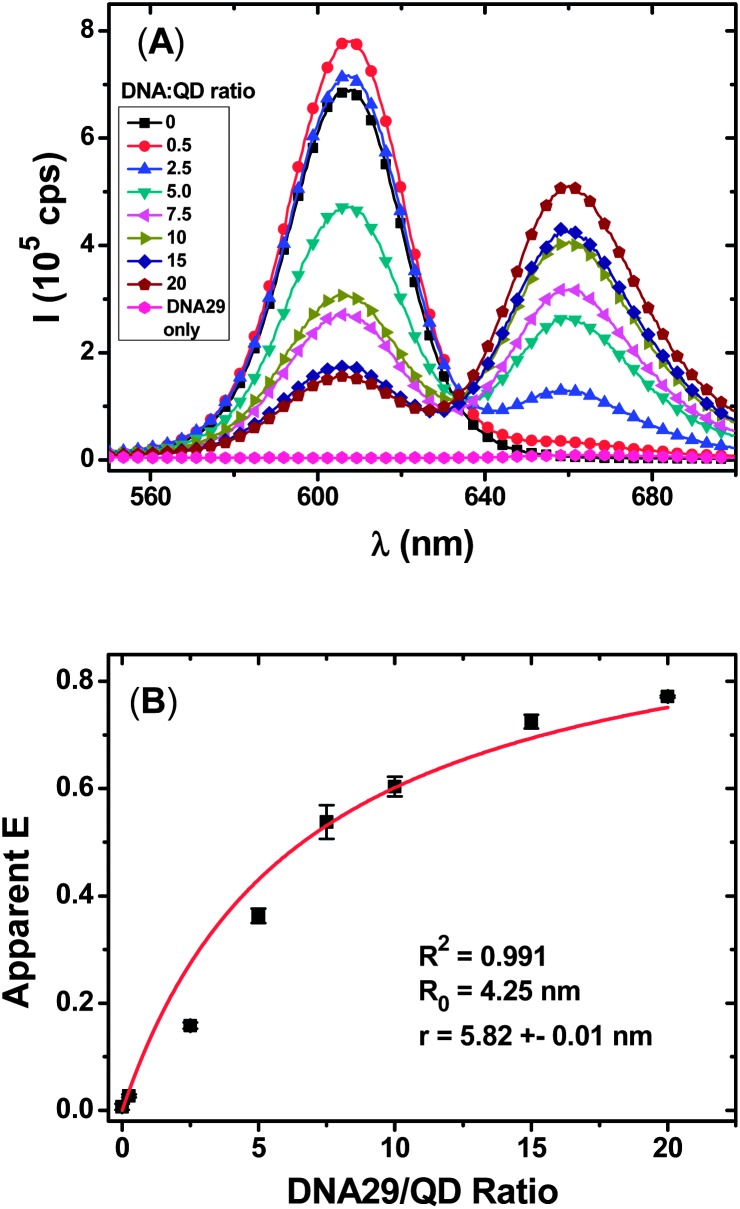
(A) Fluorescence spectra of QD–TBA_20_ (2 nM) hybridised with different molar equivalents of DNA29 with a 7 μM His_6_–Cys peptide. The DNA29 only curve has a concentration of 60 nM. (B) A plot of the apparent *E* as a function of DNA29/QD molar ratio; data were fitted using eqn (4), yielding a *r* value of 5.82 ± 0.01 nm.

Fig. 1A clearly shows that with the increasing molar ratio of the DNA29 : QD–TBA_20_, the QD fluorescence is quenched while the Atto647N FRET signal is increased progressively, suggesting efficient FRET between the QD and the Atto647N dye. Moreover, the resulting *E* and DNA29/QD molar ratio can be fitted well by the single-QD donor FRET with multiple identical acceptor models (*R* ^2^ = 0.991) with a relatively short donor–acceptor distance *r* of 5.82 ± 0.01 nm. This result confirms that the CFCC clicked QD–DNA conjugate FRET system is indeed compact, and moreover, all Atto647N labels on the DNA29 strands are bound to the QD at an identical spatial separation (the same *r* value).

### Detection of DNA29 using the CFCC clicked QD–TBA_20_

The sensitivity of the QD–TBA_20_ based FRET sensor in detecting complementary DNA probes was further evaluated by using DNA29. Hybridization of DNA29 with the QD–TBA_20_ should bring the Atto647N dye (acting as the FRET acceptor) in the close proximity to the QD, leading to the QD sensitized Atto647N FRET signal upon excitation of the QD. An advantage of the FRET based signal readout over other approaches (*e.g.* donor quenching or life time change) is its ratiometric signal, which can be effectively insensitive to signal fluctuation and instrument noise, allowing for more reliable and accurate detection.^2^ Moreover, since FRET only happens over short distances (*ca.* < 10 nm), any free, unbound species will be too far to participate in FRET with the QD donor and hence undetected, allowing for convenient probe detection to be carried out in a separation-free format.^2,3n^ Interestingly, the QD–TBA_20_ (*C*_QD_ = 2 nM) fluorescence in PBS (10 mM sodium phosphate, 150 mM NaCl, pH 7.40) was found to be enhanced significantly after treatment with a cysteine–histidine_6_ short peptide and/or bovine serum albumin (BSA, see ESI, Fig. S3A†), presumably because these molecules can bind or adsorb onto the QD–TBA_20_ to enhance the QD fluorescence QY as reported previously.^3*a*^ They may also adsorb onto sample tubes to reduce the non-specific adsorption and/or salt-induced aggregation of the QD. Moreover, the added peptide/BSA was also found to improve the FRET efficiency of the QD–TBA/DNA29 system considerably (see ESI, Fig. S3B†), and the effect became saturated at ∼7 μM. With the peptide/BSA being added, the hybridized QD–TBA_20_/DNA29 FRET system was found to be highly stable, no significant change of the QD fluorescence or Atto647N FRET signals was observed after being stored for 18 h in PBS (see ESI, Fig. S4†). This is important for biosensing, allowing the experiments to be carried out at ease without the need to worry about the stability of the sensor (from our own experience, most small-molecule ligand capped QDs, including glutathione, 3-mercaptopropinoic acid (MPA) and DHLA, showed rather limited stability in PBS, and therefore all sensing measurements should be performed within 1 h after target addition to avoid a significant decrease of the QD fluorescence).^3l–n,4d,e^ All subsequent sensing experiments were carried out with 7 μM added peptide/BSA on a conventional fluorimeter with a low QD concentration of 2 nM.

**Fig. 2:**
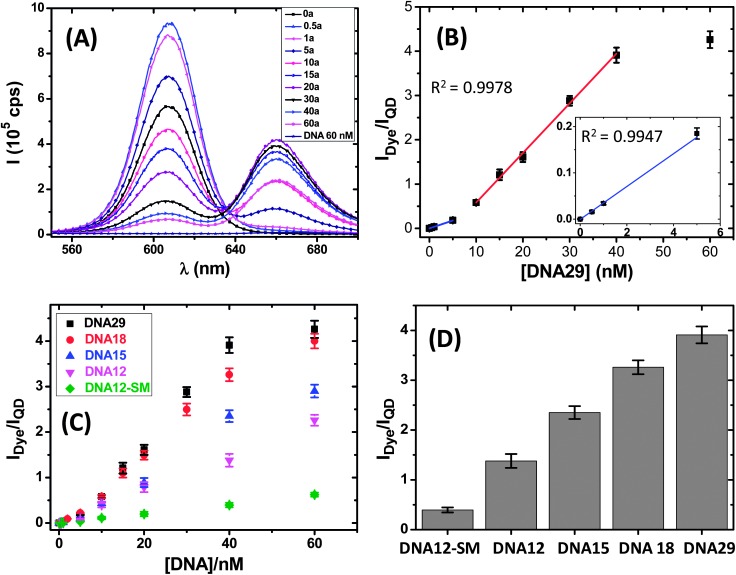
(A) Fluorescence spectra of the QD–TBA_20_ (*C*_QD_ = 2 nM) after hybridization with different amounts of DNA29 for 2 h in PBS excited at 450 nm, the *λ*_abs_ minimum of Atto647N. (B) A plot of the integrated donor/acceptor fluorescence ratio, *I*_Dye_/*I*_QD_, as a function of [DNA29]. The data were fitted to a two-stage linear relationship with fitting parameters of *y* = –0.539 + 0.1121*x*, *R* ^2^ = 0.9978 over 10–40 nM, and *y* = –0.000395 + 0.0354*x*, *R* ^2^ = 0.9947 over 0–5 nM (shown in the inset), in which the detection limit is based on. (C) Plot of the *I*_Dye_/*I*_QD_ ratios as a function of concentration of different length complementary DNA probes. (D) The *I*_Dye_/*I*_QD_ ratios for different length DNA probes at 40 nM.

Fig. 2A shows that in general the QD fluorescence (peaking at ∼605 nm) was quenched progressively together with a concurrent simultaneous significant increase of the Atto647N FRET signal (peaking at ∼665 nm) with the increasing DNA29 concentration, [DNA29], suggesting efficient QD-sensitised dye FRET from hybridisation of the DNA29 with QD–TBA_20_. A careful examination of the Atto647N emission spectra over the 640–700 nm range revealed that the Atto647N emission obtained from direct excitation of 60 nM DNA29 was actually weaker than that of the QD-sensitized FRET signal for 0.25 nM DNA29 (see ESI, Fig. S5†), suggesting that the QD sensitized FRET is at least 240 times as efficient as direct excitation. To our knowledge, this has been the highest ratio of FRET-sensitized signals over that of direct excitation for the QD-FRET systems reported so far (most reported ratios in the literature typically ranged from ∼2.5 to 40).^2,3^ This is presumably because *λ*_EX_ = 450 nm, corresponding to the *λ*_abs_ minimum of the Atto647N, was used here to minimise the direct excitation of the Atto647N acceptor. Moreover, the CFCC conjugated QD–TBA_20_ here retained a much higher QY of the QD than those prepared *via* EDC–NHS coupling (see ESI, Fig. S1†), as a result, the sensing experiments were able to be performed at 2 nM QD, ∼10 to 500 fold lower than those reported previously (see Table 2).^2,3^ Such a high FRET-sensitised signal over the direct excitation background is highly advantageous for biosensing, which can effectively eliminate the need for background correction from direct acceptor excitation, making data analysis easy and straightforward.

**Table 2:** Comparison of the sensitivity and specificity of some QD-FRET based sensors and other direct DNA detection techniques without probe amplification^*a*^

Detection system	*C*_QD_ (nM)	Target/length	Specificity	SNP DR	LOD (nM)	Ref.
QD-BRET	20	DNA/22 mer	?	?	20	3*o*
QD-FRET	1000	DNA/19 mer	<2	No	40	3*g*
QD-FRET	60	DNA/24 mer	<2	No	12	3*g*
QD-FRET	100	DNA/25 mer	∼3	No	200	3*p*
QD-FRET	?	DNA/18–32 mer	2–3	No	∼5	3*q*
QD-FRET	100	DNA/30 mer	Yes	No	∼1	3*l*
QD-FRET	4	DNA/30 mer	34	No	0.5	3*m*
QD-FRET	2	DNA/12–29 mer	816	3.3 (12 mer)	0.091 (29 mer)	This work
Microcantilever	/	DNA/12 mer	?	?	75	12*a*
Microcantilever	/	DNA/12 mer	∼3	?	∼0.010	12*b*
Electrochemical	/	DNA/24 mer	?	?	0.01	12*c*
Electrochemical	/	DNA/34 mer	Yes	1.5–2.0	0.05	12*d*
Direct SPR	/	DNA/16 mer	?	?	10	12*e*
Direct QCM	/	DNA/509 mer	?	?	10	12*f*

^*a*^BRET: bioluminescence resonance energy transfer; LOD: limit of detection; specificity: the FRET ratio between the full- and non-complementary DNA probes; SNP DR: SNP discrimination ratio.

Despite that the QD fluorescence does not always follow a simple trend of progressive quenching with the increasing [DNA29], especially at low [DNA29] shown in Fig. 2A, possibly due to a slight increase of the QD QY as DNA29 is hybridized, this has no impact on the ratiometric based data analysis employed here. In fact, the ratio of the integrated fluorescence intensity between the acceptor and the donor, *I*_Dye_/*I*_QD_ (see the ESI† for the detailed calculation method)^3*l*^ displayed a two-stage linear dependence with the increasing [DNA29]: a slow increasing phase over low [DNA29] (0–5 nM, slope: 0.0353 nM^–1^, *R* ^2^ = 0.9947) and a more rapid phase at higher concentrations (10–40 nM, slope: 0.1121 nM^–1^, *R* ^2^ = 0.9978, Fig. 2B). As [DNA29] was increased to above 40 nM, the *I*_Dye_/*I*_QD_ value showed a little further increase, suggesting that the hybridization reached saturation. Thereafter, any extra added DNA29 strands were unable to hybridize with the QD–TBA_20_ and would remain free. Since FRET only takes place over short distances (*ca.* < 10 nm), such free DNA29 strands are unable to participate in the FRET process and hence undetected. Note here that 40 nM corresponds to the total [TBA] in the 2 nM QD–TBA_20_ conjugate, suggesting that all TBAs conjugated to the QD are functional and available for hybridization. The detection limit (DL) for DNA29, based on 3 times the standard deviation/slope of linear calibration over the lower concentration range (3*σ*/slope), is estimated as ∼91 pM,^3*h*^ making it one of the most sensitive QD-FRET based sensors for direct DNA quantification without probe pre-amplification using conventional fluorescence spectroscopy (see Table 2). Moreover, this level of sensitivity is also comparable to or better than many other sensitive direct DNA detection methods without probe pre-amplification, such as the optimised, sensitive microcantilever sensors (∼10 pM),^12*b*^ electro-chemical detection (10 pM),^12*c*^ surface plasmon resonance (SPR, 10 nM)^12*d*^ and quartz crystal microbalance (QCM, 10 nM)^12*e*^ detection (see Table 2 for details).

Theoretically, a linear correlation between the *I*_Dye_/*I*_QD_ and the probe concentration, [DNA29], is expected if all introduced DNA probes are hybridized with the QD–TBA_20_ at identical positions (the same QD–dye distance, *r*).^13^ The excellent linear relationship observed here clearly confirmed that all DNA29 strands were hybridized with the QD at identical spatial separation between the QD donor and the dye acceptor. This result is also in good agreement with the earlier FRET analysis where *E* can be fitted very well (*R* ^2^ = 0.991) by the single-donor FRET with multiple identical acceptor models.^2^ The two-stage linear dependence observed here may indicate two different phases of DNA hybridization: the slower increase over the 0–5 nM range is likely due to incomplete hybridization of DNA29 with the QD–TBA_20_, arising presumably from the low, sub-*K*_d_ levels of [DNA29], whereas the faster increase over the 10–40 nM range may be attributed to more effective, complete binding of the introduced DNA29 to the QD–TBA_20_ under such conditions. Given that *K*_d_ values of 17, 19 and 41 nM have been reported for 25,^14*a*^ 20,^14*b*^ and 12 (ref. 12*a*) mer dsDNAs, respectively, we believe such explanations here are highly plausible.

The CFCC clicked QD–DNA FRET sensor was found to be highly specific; incubation of the QD–TBA_20_ with a non-complementary probe (DNA-NC, also 3′-Atto647N labelled, see Table 1) under identical conditions (with 10 μM added BSA) produced effectively non-detectable FRET. The *I*_Dye_/*I*_QD_ ratios for the DNA29 and DNA-NC (both at 30 nM) were determined as 2.563 and 0.00314, respectively, yielding an outstanding signal discrimination ratio of 816 between the full- and non-complementary DNA probes (see ESI, Fig. S7†). The discrimination ratio here is 24–400 fold higher than previously reported QD-FRET based DNA sensors (see Table 2), demonstrating an excellent DNA sensing specificity. Moreover, the QD-FRET based DNA sensor was highly robust, it worked pretty well even in clinically relevant media, *e.g.* 10% human serum (see ESI, Fig. S8†). It should be noted that despite that numerous QD-FRET based DNA sensors have been reported in the literature, few have demonstrated the working function in serum, one of the most frequently used clinical media. These results clearly demonstrated an excellent sensing specificity and robustness of the CFCC clicked QD–DNA sensor, which we attribute to the excellent stability, and more importantly, the outstanding resistance toward non-specific adsorption of biomolecules afforded by the PEGylated capping ligands on the QD surface.^9,10^

### Detection of different length DNA probes and SNP (single-nucleotide polymorphism) discrimination

Besides offering high discrimination between complementary and non-complementary DNA probes, the CFCC clicked QD–DNA sensor can effectively discriminate complementary DNA probes of different lengths. As shown in Fig. 2C, although the *I*_Dye_/*I*_QD_ ratios increased with the increasing concentration for all probes, the rates of increase were significantly different, with DNA29 being the fastest while DNA12-SM being the slowest. A general trend here is that the *I*_Dye_/*I*_QD_ increase rate showed a positive correlation with the length of the DNA probe, *e.g.* DNA29 > DNA18 > DNA15 > DNA12 > DNA12-SM. Moreover, DNA18 also showed a two-stage *I*_Dye_/*I*_QD_–[DNA] linear increase similar to that for DNA29, while for DNA15 and DNA12, this became much less clear, and DNA12-SM effectively displayed a single linear dependence. Such differences may reflect the different *K*_d_s of the different length probes toward the common TBA target: only those with *K*_d_s that span across the [DNA] range studied here may display two-stage dependence.

The slopes of the rapidly increasing *I*_Dye_/*I*_QD_ phase (over 10–40 nM range) were found to be 0.112, 0.091, 0.062, 0.036 and 0.011 nM^–1^ for DNA29, DNA18, DNA15, DNA12 and DNA12-SM, respectively. Therefore the slope of the *I*_Dye_/*I*_QD_ increase rate for the DNA29 is ∼3 times that of DNA12, while that for DNA12 is a further ∼3.3 times that of DNA12-SM, the same length (12 mer) probe containing just a single base mismatch with TBA, equivalent to single nucleotide polymorphism (SNP). The CFCC clicked QD–DNA sensor can therefore offer a SNP discrimination ratio of ∼3.3 for the 12 mer DNA probes. Similar levels of probe length dependence and SNP discrimination ratio (*ca.* > 3 between DNA12 and DNA12-SM) were also obtained from the *I*_Dye_/*I*_QD_ ratios at 40 nM probe concentration (Fig. 2D). More interestingly, the discrimination between DNA12 and DNA12-SM was found to be unaffected by the presence of complex media, such as 10% human serum. In fact, the discrimination ratio actually increased to 6.1 (against ∼3 in PBS, see ESI, Fig. S9†), demonstrating good potential for SNP based clinical diagnosis. It should be noted that despite several QD-FRET based DNA sensors have been reported in the literature, most of which displayed rather low discrimination ratios between full- and non-complementary DNA probes, few have displayed the SNP discrimination ability (see Table 2). Since SNPs are known to be closely associated with a number of important human diseases, such as cancer, neurodegenerative diseases and diabetes, *etc.*,^15^ the excellent specificity, sensitivity and robust SNP discrimination ability in complex media may make the CFCC clicked QD-DNA sensor potentially suitable for clinical applications.

### Detection of unlabelled DNA probes

The ability of detecting unlabeled DNA probes is more useful for potential clinical applications, avoiding the need for the probe labelling step which can be complex, expensive and sometimes even impossible. In this regard, a new DNA displacement assay is developed here: a longer unlabeled probe (*e.g.* DNA29-NL, with the same sequence as DNA29 but without the Atto647N label) that forms more stable duplex with TBA can effectively displace a shorter labelled DNA (*e.g.* DNA12-SM, acting as a FRET reporter) pre-hybridized with the QD–TBA_20_, leading to a decreased FRET as the unlabelled probe readout signal (Scheme 1C).

**Fig. 3:**
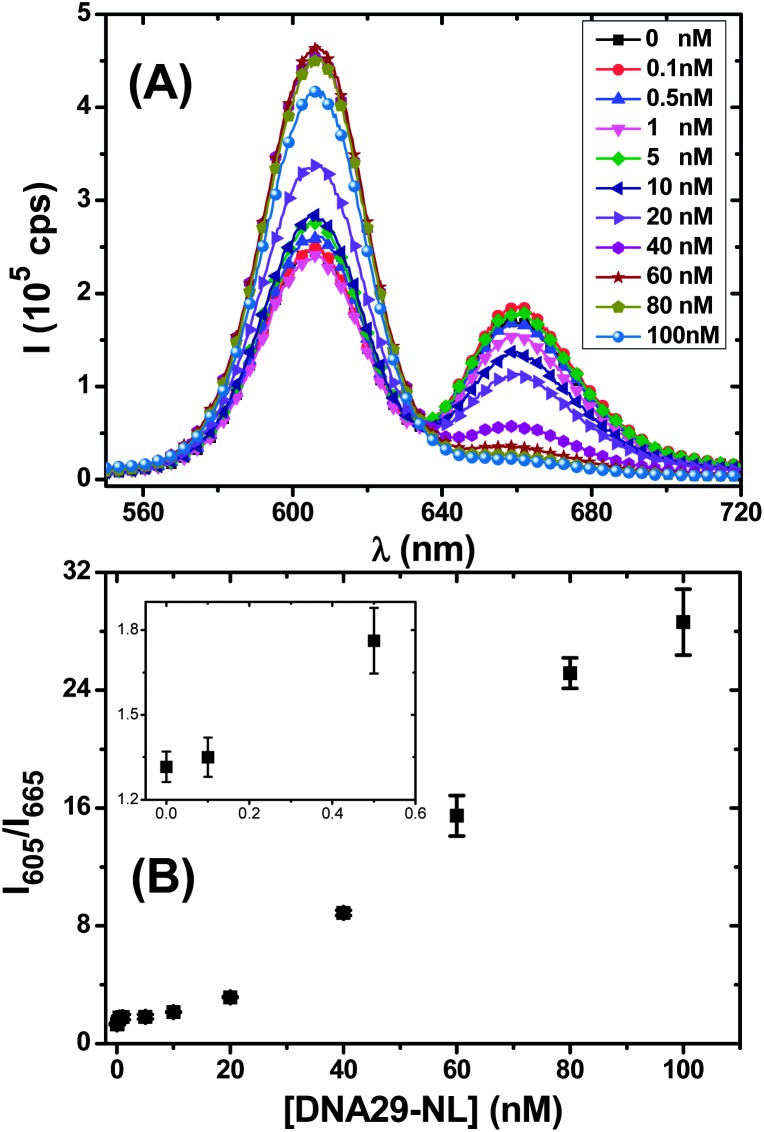
(A) Typical fluorescence spectra of QD–TBA_20_ (2 nM) pre-hybridized with DNA12-SM (60 nM) after addition of different [DNA29-NF] for 2 h (B) A plot of the corresponding fluorescence intensity ratio at 605 and 665 nm (*I*_605_/*I*_665_) as a function of [DNA29-NF], the inset shows *I*_605_/*I*_665_ responses in the sub-nM range.

Fig. 3 reveals that this is indeed feasible, where the Atto647N FRET signal at ∼665 nm was almost diminished accompanied by a concurrent significant recovery of the QD fluorescence at 605 nm as the [DNA29-NL] was increased, suggesting a successful displacement of the DNA12-SM reporter strand by the DNA29-NL, leading to a significant increase (∼21 fold) of the *I*_605_/*I*_665_ ratio (from 1.32 ± 0.06 to 28.6 ± 2.2 as the [DNA29-NL] increased from 0 at 100 nM, see Fig. 3B). Interestingly, replacing the DNA12-SM with DNA12 as the FRET reporter strand led to a much smaller increase of the *I*_605_/*I*_665_ ratio under identical conditions (from 0.66 ± 0.02 to 3.48 ± 0.09, an increase of ∼5.3 fold, see ESI, Fig. S10† for details), suggesting that a high stability difference between the reporter and the probe DNAs for the common target is key to achieve efficient reporter strand displacement and hence the greatly increased *I*_605_/*I*_665_ ratio. The *I*_605_/*I*_665_ response curve as a function of [DNA29-NL] was found to be non-linear (Fig. 3B), where 500 pM [DNA29-NL] produced a signal consistently above the background (Fig. 3B, inset), suggesting that this sensor can readily detect 500 pM DNA29-NL without probe amplification. Therefore this signal-on DNA sensing approach developed here can readily detect ∼500 pM unlabelled DNA probes together with a maximum ratiometric signal enhancement of ∼21 fold, which is already competitive against some other more established DNA sensing approaches, such as molecular beacons (*ca.* 10–20 fold signal enhancement with single-quenchers, nM sensitivity)^11b,c^ and a recently optimised electrochemical DNA sensor (*ca.* 8-fold).^12*d*^ An advantage of our approach here is its ratiometric signal, which is insensitive to instrument noise and signal fluctuation, allowing more reliable target detection. In addition, the DNA displacement assay was found to work equally efficiently in complex media, such as PBS with a large excess of BSA (10 μM) and in 10% human serum, suggesting that it may have good potential for clinical applications.

### Detection of unlabelled proteins

**Fig. 4:**
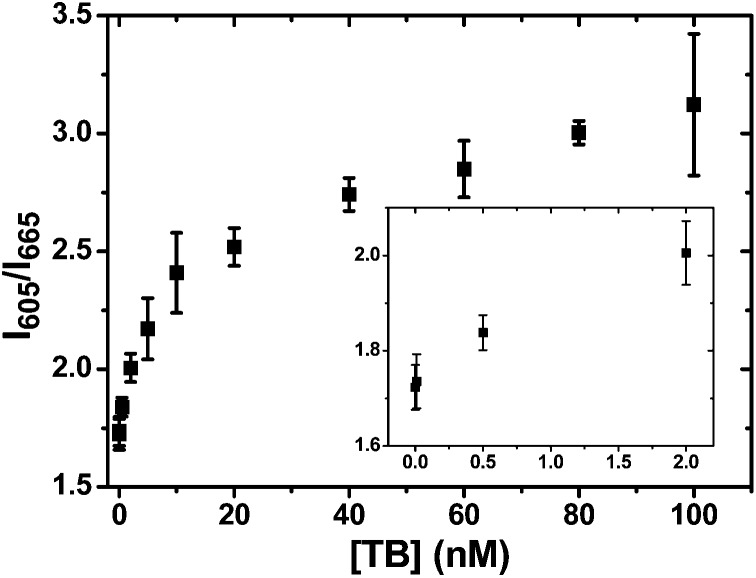
Label-free detection of thrombin using the CFCC clicked QD–DNA aptamer sensor using 2 nM QD–TBA_20_ pre-hybridized with DNA12-SM (60 nM) in PBS containing 20 μM BSA. A typical calibration curve showing the *I*_605_/*I*_665_ ratio as a function of thrombin concentration [TB] (inset: the response over 0–2 nM of [TB]).

The CFCC clicked QD–TBA_20_ can be readily extended for label-free protein sensing *via* the anti-thrombin DNA aptamer sequence encoded within the TBA sequence, where the formation of thrombin (TB)–TBA complex can effectively displace the pre-hybridised reporter DNA12-SM, leading to the FRET decrease (and hence an increase of the *I*_605_/*I*_665_ ratio, see Scheme 1D). Fig. S11 (ESI†) reveals that this was indeed true, where the Atto647N FRET signal gradually decreased while the QD fluorescence increased concurrently as the target [TB] was increased, leading to the increased *I*_605_/*I*_665_ ratio (see Fig. 4). The maximum *I*_605_/*I*_665_ ratio obtained at 100 nM TB here (∼3.1) was not as high as that obtained in DNA29-NF detection (∼29), suggesting that the TB binding here is less efficient in displacing the DNA12-SM reporter strands from the QD–TBA_20_ conjugate as compared to DNA29-NF. Given that the binding affinity between the 29 mer anti-TB aptamer and TB (*K*_d_ ∼ 0.5 nM)^11*a*^ is as strong as that of the TBA/DNA29 duplex (most likely to be in the low nM range as described above) here, the relatively low efficiency in displacing the reporter strands observed for TB here is therefore attributed to the significantly greater size of the TB–aptamer complex as compared to the TBA/DNA29 duplex, leading to steric hindrance and reduced accessibility for TB binding on the QD–DNA conjugate, especially under high [TB] conditions. Similar to the DNA29-NL based displacement assay above, a non-linear response curve between the *I*_605_/*I*_665_ signal and [TB] was also observed (Fig. 4). Moreover, the amplified response curve over the 0–2 nM [TB] range revealed that 500 pM [TB] produced a signal consistently above the background (Fig. 4, inset), suggesting that the CFCC clicked QD–DNA aptamer sensor can detect 500 pM TB directly without target pre-amplification. This sensitivity achieved here is among those of the most sensitive QD-FRET based label-free TB sensors using direct target detection without pre-amplification (see ESI, Table S1†). Moreover, this sensitivity is also comparable to those of other more established electrochemical sensing methods for TB detection (∼1 nM detection limit).^12*g*^

## Conclusion

In summary, we have successfully developed a reliable CFCC approach for the preparation of a compact and stable QD–DNA/aptamer conjugate that can retain the native fluorescence QY of the parent QD. The resulting QD–DNA conjugate is relatively compact and can effectively resist non-specific adsorption. It has been successfully exploited for robust, sensitive and ratiometric quantitation of specific DNA probes directly with pM sensitivity even in complex media, such as 10% human serum. This QD–DNA FRET sensor has offered an excellent signal discrimination (>800 fold) between the full- and non-complementary DNA probes, which is the highest for the QD-FRET based sensors. Moreover, it can discriminate between the perfect-match and the SNP targets in 10% serum. The sensor has also been exploited for sensitive label-free detection, at the pM level, of thrombin *via* the anti-thrombin aptamer sequence encoded in the QD–DNA conjugate. This QD–DNA/aptamer sensor can be readily extended for detection of other DNA and protein targets by clicking other specific DNA/aptamer sequences against such targets.^16^ Given its high stability, specificity, robustness and sensitivity, the CFCC clicked QD–DNA/aptamer sensor appears to have good potential for a wide range of biosensing and diagnostic applications.
