# Supplementary material for: Robust and specific ratiometric biosensing using a copper-free clicked quantum dot–DNA aptamer sensor
Source: Nanoscale. 2013 Sep 11;5(21):10307–15. doi: 10.1039/c3nr02897f (PMC3814187; doi:10.1039/c3nr02897f)
Supplement: Supplementary file 1 [file NR-005-c3nr02897f-s001.pdf]

Electronic Supporting Information (ESI)

Robust, specific ratiometric biosensing using a copper-free clicked quantum dot-DNA aptamer sensor

Haiyan Zhang,[a] Guoqiang Feng,[b] Yuan Guo,[a,]* and Dejian Zhou[a,]*

[a] *School of Chemistry and Astbury Centre for Structural Molecular Biology, University of Leeds, Leeds LS2 9JT, United Kingdom. E-mail: y.guo@leeds.ac.uk (YG); d.zhou@leeds.ac.uk (DZ)*

[b] *College of Chemistry, Central China Normal University, 152 Luoyu Road, Wuhan 430079, P. R. China*

Experimental Section

A) Materials

CdSe/ZnS core/shell QD (EviDot ED-C11-TOL-600, emission peak ~600 nm, crystal diameter ~ 5 nm, quantum yield ~20%) was purchased from Evident Technologies (Troy, New York, USA). The QD was supplied as toluene solution capped with TOP (trioctylphosphine) and TOPO (trioctylphosphineoxide) ligands, abbreviated as TOPO-QD. Poly(ethylene glycol) 600 (PEG600), methanesulfonyl chloride (>99.7%), sodium azide (>99.5%), tri-phenylphosphine (>98.5%), dicyclohexylcarbodiimide (DCC, >99%), dimethylaminopyridine (DMAP, >99%), thioctic acid (TA, >99%), tris(2-carboxyethyl)phosphine hydrochloride (TCEP, >98%), triethylamine (>99%), bromoform (>99%), cycloheptene (>97%), potassium tert-butoxide (>98%), anhydrous pentane (>99%), silver perchlorate (>99%), methyl glycolate (>98%), 1,8-diazabicyclo[5.4.0]undec-7-ene (DBU, >98%), *N*-(3-dimethyl-aminopropyl)-*N*-ethylcarbodiimide hydrochloride (EDC.HCl, 99%), N-hydroxysuccinimide (NHS, 98%), sodium bicarbonate (>99.5%), glutathione (>98%), DNAse and RNAse free NaCl (>99%), Na_2HPO_4 (>99%), thrombin and all other chemicals and reagents were all purchased from Sigma-Aldrich (Dorset, UK). All chemical and solvents are purchased commercially from Sigma-Aldrich (Dorset, UK) and used without further purification unless stated otherwise. Solvents were obtained from Fisher Scientific (Loughborough, UK) and used as received.

All moisture-sensitive reactions are performed under nitrogen atmosphere using oven-dried glassware. All solvents are dried by innovative technology prior to use, or are taken from sealed-bottles under nitrogen atmosphere. Evaporations are performed under reduced pressure on a rotary evaporator. Reactions are monitored by TLC on silica gel 60 F254 plates on aluminium and stained by iodine. Flash column chromatography was performed on silica gel 60 A (Merck grade 9385). ^{1}H and ^{13}C NMR spectra of the samples are recorded on Brucker DPX300 (500 MHz for ^{1}H, 125 MHz for ^{13}C) in $CDCl_3$. All chemical shifts are reported herein in ppm and the coupling constants are reported in Hertz.

Ultra-pure water (resistance >18.2 MΩ.cm) purified by an ELGA Purelab classic UVF system, was used for all experiments and making buffers. All DNA oligonucleotides were commercially custom synthesized and purified by IBA GmbH (Göttingen, Germany) and used directly without further purification. The amine modified and non-

modified DNAs were HPLC purified while Atto-647N labelled DNAs were double-HPLC purifed. Their sequences and abbreviations are given in Table 1 in the main manuscript.

B) Instrument and Methods

All UV-vis absorption spectra of the quantum dot and oligonucleotides were recorded on a Varian Cary 50 bio UV-Visible Spectrophotometer over the range of 200-800 nm using 1 mL curtz cuvette with an optical path length of 1 cm. All fluorescence spectra were measured on a Spex Fluoro Max-3 Spectrofluorometer using a 0.70 mL quartz cuvette under a fixed excitation wavelength of 450 nm which corresponds to the absorption mimimum of the Atto647N to minimize direct excitation of the acceptor. An excitation and emission bandwidths of 5 nm as a scan rate of 120 nm/min over 550-720 nm range were used. All centrifugations were carried out on a Thermo Scientific Heraeus Fresco 21 microcentrifuge using 1.5 mL microcentrifuge tubes at room temperature (unless stated otherwise). The QD purification was performed by Amicon ultra-centrifugal filters with a cut-off MW of 30,000.

C) Experimental Procedures[1]

C1) Synthesis of DHLA-PEG600-N_3 capped CdSe/ZnS core/shell QD, QD-DHLA-PEG600-N_3.

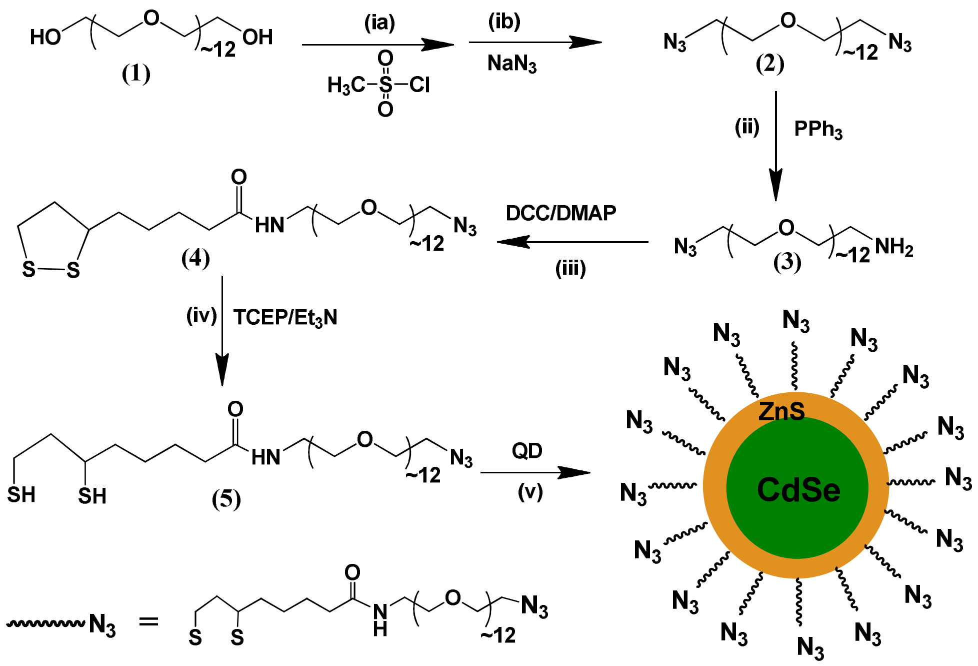

Scheme S1. The synthetic route to DHLA-PEG600-N_3 capped CdSe/ZnS core/shell QD: QD-DHLA-PEG600-N_3. The reaction conditions are: (ia) MsCl, Et_3N, THF; (ib) NaN_3, $NaHCO_3$, H_2O; (ii) PPh_3, EtOAc and 1 M HCl; (iii) thioctic acid, DCC/DMAP, CH_2Cl_2; (iv) TCEP, Et_3N, EtOH/H_2O; (v) N_2, $CHCl_3$, RT.

Step 1: Synthesis of N_3-PEG_{600}-N_3 (2)

Polyetheylene glycol with an average molecular weight of 600 (PEG600) (30 g, 50 mmol), THF (50 ml) and methanesulfonyl chloride (13.2 g, 115 mmol) were added in a 250 ml two-necked round-bottomed flask equipped with an addition funnel, septa and a magnetic stirring bar. Triethylamine (17.2 ml, 123 mmol) was added to the addition funnel. The reaction mixture was purged with nitrogen and cooled to 0 °C in an ice bath. Triethylamine was then added dropwisly to the reaction mixture through the addition funnel (addition takes ~30 min). After that, the reaction mixture was warmed up gradually to room temperature (~20 °C) and stirred overnight. The product was checked by TCL on silica gel with $CHCl_3$:MeOH =10:1 (vol/vol) as elution solvent, R_f(MsO–PEG_{600}–OMs) = 0.62, R_f (HO–PEG_{600}–OH) = 0.36. The mixture was then diluted with H_2O (66 ml) and $NaHCO_3$ (4.13 g, 0.049 mol) was added. The resulting mixture was transferred to a separator funnel and extracted with $CHCl_3$ (60 ml × 3). The combined organic phase was evaporated by driness on a rotary evaporator, yileding slightly yellowish oil, 40g, with ~90% yields.

The product (40 g), sodium azide (10 g, 0.154 mol), THF (50 ml), H_2O (50 ml) and $NaHCO_3$ (0.5 g) were then added to two-necked round-bottomed flask equipped with a distilling head with a round-bottomed flask as a solvent trap. The solvent trap was cooled with an ice-bath. The biphasic reaction mixture was heated under N_2 to distill off the THF. The reaction mixture was then refluxed for ~8 hrs (or overnight). After the reaction mixture was cooled to room temperature, it was transferred to a separator funnel. The product was extracted with $CHCl_3$ (100 ml × 5). The product was checked with TCL using $CHCl_3$: MeOH =10:1 (vol/vol) as elution solvent, R_f (N_3-PEG_{600}-N_3) = 0.75. The combined organic layers were dried over Mg_2SO_4 (~20 g, for ~30 min) with stirring. The Mg_2SO_4 was filtered off and the dried organic layer was evaporated to dryness on a rotary evaporator. A pale brown oil (33.21g, yield: 97%) was obtained . ^{1}H NMR (500 MHz, $CDCl_3$): d 3.62–3.71 (m), 3.40 (t, 4H, *J* =5.0 Hz)

Step 2: Amine transformation of one terminal azide group, synthesis of N_3-PEG_{600}-NH_2 (compound 3)

N_3–PEG_{600}–N_3 (8.012g, 11.7 mmol), EtOAc (60 ml) and 1 M HCl (27 ml, 27 mmol) was added in a 250 ml two-necked round-bottomed flask equipped with an addition funnel, septa and a magnetic stirring bar. Triphenyl-phosphine (3.36 g, 12.8 mmol) dissolved in 45 ml EtOAc was transferred to the addition funnel. The reaction vessel was purged with N_2 and cooled to 0 °C in an ice-bath while stirring. The triphenylphosphine solution was then added dropwisely under N_2. The temperature was maintained to below 5 °C during the addition. Once the addition was completed, the reaction mixture was gradually warmed up to room temperature and stirred for ~8 h (or overnight) under N_2. The reaction mixture was then transferred to a separatory funnel and the biphasic solution was separated. The aqueous layer was collected and washed with EtOAc (60 ml × 2). The aqueous layer was transferred to a round-bottomed flask with a magnetic stirring bar. The solution was cooled in an ice bath. KOH (13.5 g) was then added slowly to the aqueous solution and the mixture was stirred until the KOH is dissolved. The aqueous solution was transferred into a separatory funnel and extracted repeatedly with EtOAc (60 ml × 5). The combined organic layer was dried over $MgSO_4$ (20 g for ~20 min) with stirring. After filting off $MgSO_4$, the solvent was evaporated to dryness on a rotary evaporator, yielding a light yellow oil, 6.5 g, yield: ~80%. R_fs ($CHCl_3$:MeOH = 5:1, vol/vol) for N_3–PEG_{600}–NH_2 = 0.09, N_3–PEG_{600}–N_3 = 0.8. ^{1}H NMR (500 MHz, CDCl3): d 3.6–3.9 (m), 3.52 (t, 2H, *J*= Hz), 3.40 (t, 2H, *J*= 4.8 Hz), 2.87 (t, 2H, *J* =5.0 Hz)

Step 3: preparation of TA-PEG$_{600}$-N$_3$ (Compound 4)

N_3-PEG$_{600}$-NH_2 (6.5 g, 9.8 mmol), 4-(N,N-dimethylamino)pyridine (0.2407 g, 1.977 mmol), N,N'-dicyclohexyl-carbodiimide (2.07 g, 10.07 mmol) and CH_2Cl_2 (60 ml) were added into a 250 ml round-bottomed flask equipped with a magnetic stirring bar and an addition funnel. The mixture was kept at 0 °C in an ice bath. Thioctic acid (2.02 g, 9.8 mmol) dissolved in 32 ml of CH_2Cl_2 was transferred to the addition funnel. Thioctic acid solution was added dropwisely over 30 min under N_2 while stirring. After the addition was complete, the reaction mixture was allowed to warmed up to room temperature gradually, and the mixture was stirred overnight. The mixture was then filtered off through celite and the celite plug was rinsed with $CHCl_3$. The solvent was evaporated on a rotary evaporator. 100 ml H_2O was added to the residue and the mixture was washed with diethyl ether (100 ml × 2). The aqueous layer was then saturated with $NaHCO_3$. The product was extracted with $CHCl_3$ (100 ml × 3). The combined organic layers were dried over $MgSO_4$ (for 20 min). After filtering off the $MgSO_4$, the solvent was evaporated on a rotary evaporator. The residue was then purified on a silica gel chromatograph (5.0 cm i.d. × 20 cm length) using 20:1 (vol/vol) CHCl3: MeOH as the eluent solvent. Each fraction was checked by TLC and those with the pure product fraction were combined ($CHCl_3$: MeOH =10:1 (vol/vol), R_f (TA-PEG$_{600}$-N$_3$) = 0.52, R_f (TA) = 0.6, R_f (N_3–PEG$_{600}$–NH_2) = 0.04). The solvent was evaporated to dryness, giving a yellow product, weight 2.3g, yield: 28%. ^{1}H NMR (400 MHz, $CDCl_3$): d 6.42 (br s, 1H), 3.4–3.5 (m), 3.36 (t, 2H, *J*=5.0 Hz), 3.26 (t, 2H, *J*=5.0 Hz), 3.0(t, 2H, *J*=5.2 Hz), 2.8–2.9 (m, 2H), 2.2 (m, 1H), 2.0 (t, 2H, *J* = 7.4 Hz), 1.71 (m, 1H), 1.5 (m, 4H), 1.27 (m, 2H).

IR (neat): 3331; 3072; 2868; 2105; 1651; 1543; 1454; 1107 cm^{-1}.

Step 4/5: Preparation of QD-DHLA-PEG$_{600}$-N$_3$

TCEP (17.2 mg, 0.5 mmol) was dissolved in water (200 µl) and then mixed with TA-PEG$_{600}$-N$_3$ EtOH solution (0.27M, 200 µl, 0.054 mmol) for 5 mins to cleave the disulfide bond. The product DHLA-PEG$_{600}$-N$_3$ was extracted with $CHCl_3$ (200 µl × 5). The combined $CHCl_3$ layer was evaporated to dryness on a rotatory evaportor to yield a clear oil. 100 µl Et_3N was then added to the product and mixed for 5 min, after which it was evaporated on a rotary evaporator. QD$_{600}$ (40 nmol/ml in tolune, 100 µl, 4 nmol) was repeatedly precipitate, centrifuged and washed with EtOH (500 µl × 4). The final QD residue was dissolved in $CHCl_3$ (500 µl) and mixed with the DHLA-PEG$_{600}$-N$_3$ in a 20 ml round bottom flask under N_2 atmosphere at room temperature for 2 hrs. The solvent was then evaporated and residue was dissolved in EtOH. The resulting mixture was then transferred to 30,000 MW cutoff membrane and centrifuged at 7,000 × g for 3 mins followed by repeated washing with EtOH. The process was repeated three times to remove any unreacted DHLA-PEG$_{600}$-N$_3$. The final QD which has an effective MW > 30,000 and remained in the centrifuge tube was dissolved in EtOH. The quantum yield (QY) of the prepared QD-DHLA-PEG$_{600}$-N$_3$ was found to be ~6% using Rhodamine 6G (95% in ethanol) was the standard.

This journal is © The Royal Society of Chemistry 2013

C2) The synthetic route to OCT-TBA and QD-TBAn[2]

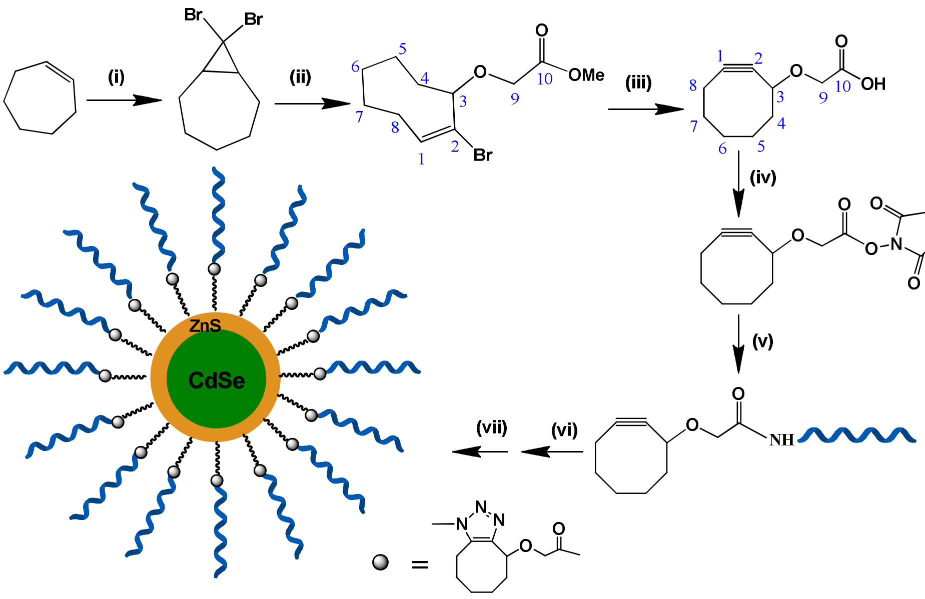

Scheme S2. The synthetic route to QD-TBA$_n$. Reaction conditions are: i) $CHBr_3$, ^{t}BuOK, anhydrous pentane, RT ; ii) $AgClO_4$, methyl glycolate, anhydrous toluene, RT ; iii) DBU, DMSO, 60°C ; (iv) NHS, DCC/DMAP; (v) $NaHCO_3$(50 mM, pH = 9.0), TBA-amine; (vi) EtOH 70%; (vii) OCT acid 1M.

Step i) Synthesis of 8,8-dibromobicyclo[5.0.1]octane.[2]

Bromoform (2.45 mL, 28.5 mmol) was added dropwisely to a vigorously stirred creamy yellow mixture of cycloheptene (1.83 g, 19 mmol) and potassium tert-butoxide (4.26 g, 38 mmol) in anhydrous pentane (5 mL) at 0°C (ice-bath) under N_2. The resulting brown mixture was then allowed to warm to room temperature and stirred overnight. Water (25 mL) was then added, and the reaction mixture was then neutralized with 1 M HCl. It was then transferred to a separation funnel and the aqueous phase was extracted with $CHCl_3$ (20 mL × 3). The combined organic phases were washed with water (20 ml × 3), dried on $MgSO_4$, and then concentrated *in vacco*. The resulting yellowish-orange oil is purified by filtration on a short silica column, eluting with hexane to afford the desired product as a colorless oil (4.1 g, 90%) . ^{1}H NMR (500 MHz, $CDCl_3$): δ (ppm) 1.12-1.24 (m, 3H), 1.30-1.41 (m, 2H), 1.67-1.74 (m, 2H), 1.80-1.92 (m, 3H), 2.23-2.28 (m, 2H). ^{13}C NMR (125 MHz, $CDCl_3$): 40.7, 34.7, 32.2, 28.9, 28.0.

Step ii) Synthesis of methyl 2-bromocyclooct-1-en-3-glycolate.[2]

Silver perchlorate (3.85 g, 18.6 mmol) was added to a solution of 8,8-dibromobicyclo[5.0.1]octane (2.5 g, 9.3 mmol) and methyl glycolate (6.35 mL, 83.9 mmol) in anhydrous toluene (5 mL) under N_2 and protected from light by an aluminium foil. The mixture was stirred at room temperature for 1.5 hrs. Silver salts are then filtered off and washed with EtOAc. The filtrate was concentrated and the residue was purified by column chromatography (10-20% EtOAc/hexane) to afford methyl 2-bromocyclooct-1-en-3-glycolate as a yellow oil (1.73 g, 67%). ^{1}H NMR (500 MHz, $CDCl_3$) : δ (ppm) 0.75-0.90 (m, 1H, H_5); 1.25-2.12 (m, 7H, $H_{4\text{-}4'\text{-}5'\text{-}6\text{-}6'\text{-}7\text{-}7'}$), 2.28-2.32 (m, 1H, H_8), 2.71-2.76 (m, 1H, $H_{8'}$), 3.75 (s, 3H, OCH_3), 3.98 (d, J = 16.5 Hz, 1H, H_9), 4.12 (dd, J = 5.0-10.0 Hz, 1H, H_3), 4.25 (d, J = 16.5 Hz, 1H, $H_{9'}$), 6.22 (dd, J = 4.0-11,5 Hz, 1H, H_1). ^{13}C NMR (125 MHz, $CDCl_3$): 26.3, 28.1, 33.4, 36.5, 39.4 ($C_{4\text{-}5\text{-}6\text{-}7\text{-}8}$), 51.8 ($C_3$), 65.5 ($C_{11}$), 84.8 ($C_9$), 131.5 ($C_2$), 133.0 ($C_1$), 170.7 ($C_{10}$).

Step iii) Synthesis of cyclooct-1-yn-3-glycolic acid.[2]

Methyl 2-bromocyclooct-1-en-3-glycolate (816 mg, 2.94 mmol) was dissolved in anhydrous DMSO (8 mL) and heated to 60°C. 1,8-Diazabicyclo[5.4.0]undec-7-ene (DBU, 7mL) was added, the resulting mixture was stirred at for 15 min and more DBU (7mL) was added. The mixture was stirred at 60°C overnight and then cooled to room temperature. Water (20 mL) was added and solution was acidified to pH 1 with concentrated HCl. The aqueous phase was extracted with $CHCl_3$ (30 mL × 3). The combined organic layers were washed with saturated NaCl solution (30 mL), dried over $MgSO_4$ and filtered. The solvent was evaporated under reduced pressure and the crude product was purified by silica gel column chromatography (10:90:1-25:75:1 EtOAc:hexane:AcOH) to yield the product as a slight yellow solid (438 mg, 82%). ^{1}H NMR (500 MHz, $CDCl_3$) δ (ppm) 1.3-2.3 (m, 10H, ring skeleton), 4.10 (d, J = 16.8 Hz, 1H, H_9), 4.25 (d, J = 16.8 Hz, 1H, $H_{9'}$), 4.38-4.40 (m, 1H, H_3), 8.85 (s, 1H, -COOH). ^{13}C NMR (125 MHz, $CDCl_3$): 20.7, 26.2, 29.6, 34.2 ($C_{4-5-6-7}$), 42.2 (C_8), 65.7 (C_3), 73.1 (C_9), 91.1 (C_1), 101.9 (C_2), 175.4 (C_{10}).

Step iv) Synthesis of cyclooct-1-yn-3-glycolic-*N*-hydroxysuccinimide ester (OCT-NHS)

Cyclooct-1-yn-3-glycolic acid (85.4 mg, 0.46 mmol), N-Hydroxysuccinimide (59 mg, 0.51 mmol), 4-dimethyl aminopyridine (DMAP) (11.8 mg, 0.102 mmol) were dissolved by dichloromethane (DCM) (2.5 mL) in a two neck flask. The mixture was stirred under N_2 for 30 min and cooled to 0°C in ice bath. A solution of *N,N'*-Dicyclohexylcarbodiimide (DCC) (107 mg, 0.52 mmol) in DCM was added dropwisely in 30 mins. The reaction solution was stirred at 0 °C for 1 hr before being warmed to room temperature and stirred at room temperature overnight. The mixture was transferred to centrifuge tube, and centrifuged at 1 kg for 10 mins to collect the clear supernatant. The precipitate was washed by 1 ml ethyl acetate twice followed centrifugation, and checked against TLC ($CHCl_3$:MeOH = 10:1) (vol/vol), R_f (Cyclooct-1-yn-3-glycolic acid) = 0.53, R_f (NHS) = 0.21, R_f (cyclooct-1-yn-3-glycolic-N-hydroxysuccinimide ester) = 0.78. The supernatants were combined and concetrated on a rotary evaporator. The resulting residue was purified by flash chromatography with 20:1 (vol/vol) $CHCl_3$:MeOH as the eluent. Each fraction was checked by TLC and the fractions containing the pure product were combined. After removing of the solvent under vacuum, the final product was obtained as a white powder (79.3 mg, yield: 56.6%) 1H NMR (500 MHz, CDCl3) δ (ppm) 1.30-2.22 (m, 8H, ring skeleton), 2.24-2.30 (m, 2H, CH_2, H_8), 2.85 (s, 4H, $2CH_2$), 4.41 (d, J = 17.0 Hz, 1H, H_9), 4.41-4.44 (m, 1H, H_3), 4.58 (d, J = 16.8 Hz, 1H, H_9).

Step v) Synthesis TBA-OCT

90 nmol $TBA-NH_2$ was dissolved in 90 µl water and then 210 µl ethanol was added to form $TBA-NH_2$ stock solution (300 µM). $TBA-NH_2$ (300 µM, 200 µl), OCT-NHS ester (0.312M, ~200 µl), $NaHCO_3$ solution (50 mM, pH = 9, 20 µl) were mixed together (OCT-NHS:H_2N-TBA molar ratio ~1000:1). The resulting solution was kept at 4 ^{o}C in a fridge for 4 hrs. After that NaOAC solution (3M, pH = 5, 40 µl), ethanol (600 µl) were added to the solution. The TBA-OCT formed white precipitate. The mixture was frozen in a freezer (~-20 ^{o}C) overnight, and centrifuged at -4 °C 20,000 × g for 30 mins, the precipitated was washed with cold ethanol 300 µl, frozen 30 mins and then centrifuged. This process was repeated for three times. The final TBA-OCT was dissolved in water 60 µl and then added ethanol 120 µl. The concentration of the TBA-OCT stock was determined by its UV absorption at 260 nm as ~ 333 µM

Step vi/vii) QD-TBA conjugation *via* copper free click chemistry

TBA-Oct (~ 30 nmol, 90 μl) was mixed QD-DHLA-PEG600-N_3 (27.87 μM, 36 μl, QY ~6.0%) in a fridge over night, leading to covalent QD-TBA conjugation *via* the CFCC. After that, OCT-COOH (0.1M, 10 μl) was then added to the mixture to quench any unreacted azide groups on the QD. This quenching step was found to be important in terms of improving the stability of the QD-TBA_n conjugate. The mixture was allowed to stand at room temperature for a further two hours. The reaction mixture was transferred to a centrifugation vial equipped with a 30,000 WMCO cutoff membrane and centrifuged at 20,000 × g followed by washing with water. An absorption spectrum was taken on the combined clear centrifuge/wash mixture and the absorbance at 260 nm was used to calculate the amount of TBAs not conjugated to the QD. This gave a value of ~ 10 nmol. Therefore ~20 nmol of TBAs have conjugated to the QD-DHLA-PEG600-N_3 (1 nmol), giving the copy number of TBAs attached to each QD of ~20, denoted as QD-TBA_{20}. The numbers of TBA strands conjugated to each QD varied from batch to batch, but were generally in the range of 15~20. The final QD-TBA_{20} was dissolved in 7 μM BSA aqueous solution for long term storage. Its quantum yield (QY) was determined using Rhodamine 6G in ethanol as the standard using the equation below:

$$QY_{QD} = QY_S \bullet (F_{QD}/F_S) \bullet (A_S/A_{QD})$$

Where QY_{QD}, F_{QD} and A_{QD} are the QY, integrated fluorescence intensity and absorbance at 480 nm of the QD sample, and QY_S, F_S and A_S are the quantum yield, integrated fluorescence intensity and absorbance at 480 nm of the standard (Rhodamine 6G, which has a QY of 95% under 480 nm exitation). The QY of the QD-TBA_{20} was found to be ~ 5.9%, effectively the same as that of the parent QD-DHLA-PEG600-N_3 (~6.0%).

C3) Preparation of glutathione QD-TBA_5 conjugate *via* EDC/NHS coupling[4]

Step 1) Preparation of glutathione modified QD

Glutathione capped QD was prepared by following our previously established procedures.[4] Briefly, TOPO-QD (80 nmol) in toluene were precipitated by EtOH. The precipitate was dissolved in chloroform (1 ml), into which was added 200μl GSH solution containing 28.4 mg GSH and 20 mg KOH in MeOH. The mixture was shaken for 30 min, and a precipitate was formed. After centrifugation, the clear supernatant was discarded and the precipitate was washed with MeOH 3 times to remove any uncapped GSH. The precipitate was dissolved in pure water to form a bright red stock solution and stored in a fridge at 4 ^oC till use, denoted as QD-GSH. Its concentration was determined from its absorbance at the exciton peak by using the Beer-Lambert law following the previously established method. Its QY was estimated as ~18% using Rhodamine 6G in ethanol (QY = 95%) as standard.

Preparation of covalently coupled QD-DNA conjugate

QD-DNA conjugation was performed by following our previous procedures.[3] Briefly, EDC (30 mg) and NHS (15 mg) were dissolved in 200 μL phosphate buffer (20 mM, pH = 5.57) into which was added 2.0 nmole of QD-GSH (223 μL, 8.98 μM), and the resulting solution was sonicated for 20 s. The mixture was allowed to stand at room temperature for 1 h to activate the carboxylic acid groups in QD-GSH into active NHS esters. After that, the solution was centrifuged (at 21,000 × g for 3 mins), and the clear supernatant was discarded. The precipitate pellet was washed carefully with water (2 × 100 μL). After that, the QD pellet was added with H_2N-TBA (200 μM 150

μL, 30 nmole), and $NaHCO_3$ buffer (50 mM, pH = 9, 100 μL) and thoroughly mixed, sonicated and then was allowed to stand overnight at 4℃. The resulting solution was then added EtOH (400 uL) and stored in a freezer for 1 h. After that, the solution was centrifuged and the clear supernatant which contained the unreacted TBA was carefully removed (checked with an UV lamp to ensure no QD was removed). The pellet was carefully washed with a mixed solution of EtOH (400 uL), water (100 uL) and 50 mM $NaHCO_3$ (100 uL). An absorption spectrum was taken on the combined clear supernatant/wash and the absorbance at 260 nm was used to calculate the amount of TBA not conjugated to the QD, giving a value of ~ 20 nmol. Therefore 10 nmol of the TBA were conjugated to the QD-GSH (2 nmol), so the number of TBA strands attached to each QD was 5, abbreviated as QD-TBA_5. Finally, the pellet was dissolved in 0.5 mL of pure water to obtain a clear QD-TBA_5 stock solution and stored in the dark at 4°C till use.

C4) Hybridization of probe DNAs to the QD-TBA_{20}.

The total volume of the hybridization solution was 400 μL, contianing a final QD concentration of 2 nM in 1× PBS (10 mM phosphate, 150 mM NaCl, pH 7.40). The hybridization reaction was carried out in batches under identical conditions, where 40 μL of the 10 × PBS, BSA and Cys-His_6 peptide, DNA probe, and the required amount of the QD-TBA_{20} and the required amount of water were added to a series of eppendorfs, and thoroughly mixed. The hybridization reaction was carried out at room temperature for 2 hrs before fluorescence spectra were taken. All fluorescence spectra were recorded on a Fluoromax-3 fluorescence spectrophotometer. The emission spectra (550 – 750 nm) were recorded under a fixed excitation wavelength of 450 nm (λ_{abs} minimium of the Atto647N acceptor to minimize its direct excitation) at a scan rate of 120 nm/min. Excitation and emission bandwidths of 5 nm were used. The quantum yield of the QD was measured using Rhodamine-6G in ethanol (95% under 480 nm excitation) as a reference. The optical densities of the QD and Rhodamine-6G solutions used were 0.05 at 480 nm.

C5) **Calculation of FRET signal**.[3]

The fluorescence intensities, I_{QD} and I_{Dye}, in this paper are the integrated fluorescence, while I_{605} and I_{670} are the fluorescence intensities at 605 and 670 nm (corresponding to the λ_{EM} maximum of the QD and dye respectively). The Atto647N FRET signal was obtained by subtracting the QD fluorescence from the total integrated fluorescence of the whole spectrum (after correction for direct excitation of Atto647N). The QD fluorescenec was calculated assuming that the QD fluorescence maintained the same shape as the QD-TBA_{20} only sample, so its integrated fluorescence is proportional to the height of the QD fluorescence peak at 605 nm where Atto647N does not fluoresce at this wavelength.

Reference:

[1] a) K. Susumu, H. T. Uyeda, I. L. Medintz, T. Pons, J. B. Delehanty, H. Mattoussi, *J. Am. Chem. Soc.*, **2007**, *129*, 13987; b) B. C. Mei, K. Susumu, I. L. Medintz, H. Mattoussi, *Nature Protocols* **2009**, *4*, 412.
[2] a) A. Bernardin, A. Cazet, L. Guyon, P. Delannoy, F. Vinet, D. Bonnaffé, I. Texier, *Bioconjugate Chem.* **2010**, *21*, 583. b) N. J. Agard, J. M. Baskin, J. A. Prescher, A. Lo and C. R. Bertozzi, *ACS Chemical Biology* **2006**, *1*, 644.
[3] D. J. Zhou, L. M. Ying, X. Hong, E. A. Hall, C. Abell, D. Klenerman, *Langmuir*, **2008**, *24*, 1659.
[4] a) Y. Zhang, H. Y. Zhang, J. Hollins, M. E. Webb, D. J. Zhou, *Phys. Chem. Chem. Phys.* **2011**, *13*, 19427; b) H. Y. Zhang, D. J. Zhou, *Chem. Commun.* **2012**, *48*, 5097.

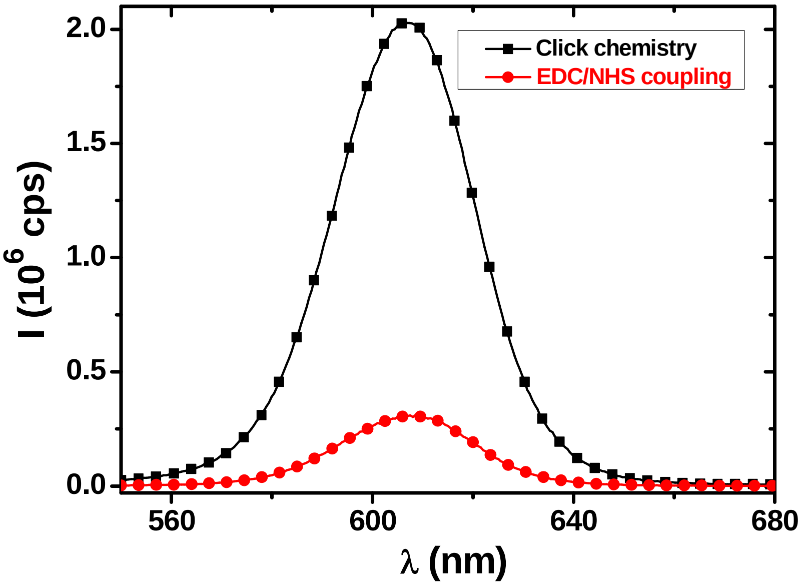

Fig. S1. Typical fluorescence spectra of the QD-TBA_{20} prepared by the Cu-free click chemistry (CFCC, 10 nM) and the QD-TBA_5 prepared *via* EDC/NHS coupling (10 nM) recorded under identical conditions. The fluorescence intensity of the CFCC prepared QD-TBA_{20} conjugate is ~ 6.4 times that of the later.

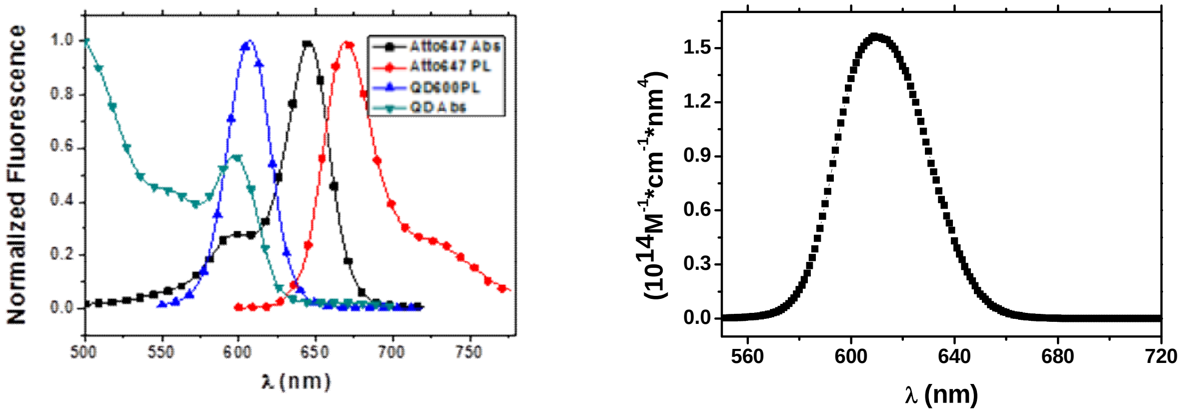

Fig. S2. Photophysical properties of the QD donor and the Atto647N acceptor. **(Left)** Normalized fluorescence emission (**Blue**)/absorption (**Green**) spectra of the QD, and the normalized fluorescenec emission (**Red**) / absorption (**Black**) spectra of the Atto647N. **(Right)** Spectra overlap function of the Atto647N and QD.

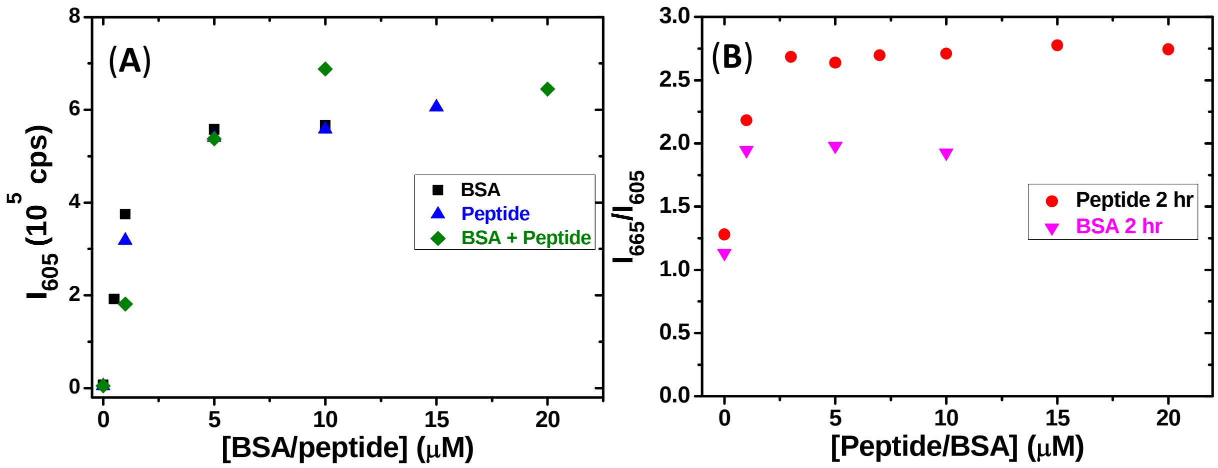

Fig. S3. (**A**) Effect of added Cys-His$_6$ peptide (Peptide) and/or bovine serum albumin (BSA) toward the fluorescence intensity of the QD-TBA$_{20}$ at 605 nm (2 nM in PBS, 10 mM phosphate, 150 mM NaCl, pH 7.40). This figure clearly show that the QD fluorescence intensity (stability in PBS) is greatly increased as the Peptide and/or BSA were introduced into the PBS buffer, possibly due to that the added Peptide/BSA can effectively bind and/or adsorb onto the surfaces of the QD as well as the microcentrifuge tubes, preventing the loss of soluble QDs due to non-specific surface adsorption (see below for the estimation of maximum amount of surface adsorbed QDs). (**B**) Effect of the added Peptide/BSA on the FRET ratio (I_{665}/I_{605}) for the QD-TBA$_{20}$ (2 nM) after hybridization with 30 nM DNA29 for 2 hrs in PBS. The added peptide and/or BSA can significantly improve the FRET ratio. This may be due to that the added Peptide/BSA can also bind to the QD to increase its quantum yield (see A. R. Clapp et al., *J. Am. Chem. Soc*. **2004**, *126*, 301), which in term leads to a greater R_0 value and hence an improved FRET ratio.

Estimation of the amount of QD adsorption

The possible maximum amount of QD adsorbed on surfaces can be simply estimated as follows: 1) assuming the QD-TBA$_{20}$s are rigid balls with a diameter of 10 nm and are closely-packed when they are adsorbed on the surface, then each QD would occupy a surface area = $3.14 \times (10/2)^2 = 86.6$ nm^2 or 8.66×10^{-13} cm^2; 2) 1 mL of the QD sample is stored in 1 mL (1cm × 1cm × 1cm) cuvette, this would give a possible surface contact area of 5 cm^2 (the top surface is ignored). Under such assumptions, then the number of surface adsorbed QDs = $5/[8.66\times10^{-13}] = 5.77 \times 10^{12}$, which is equivalent to 9.6 pmole ($5.77\times10^{12}/(6.02 \times 10^{23})$) or 9.6 nM in 1 mL sample. Therefore, it is entirely possible that all of the QDs can be completely adsorbed onto the surface when a low concetrantion of 2 nM was used in this paper.

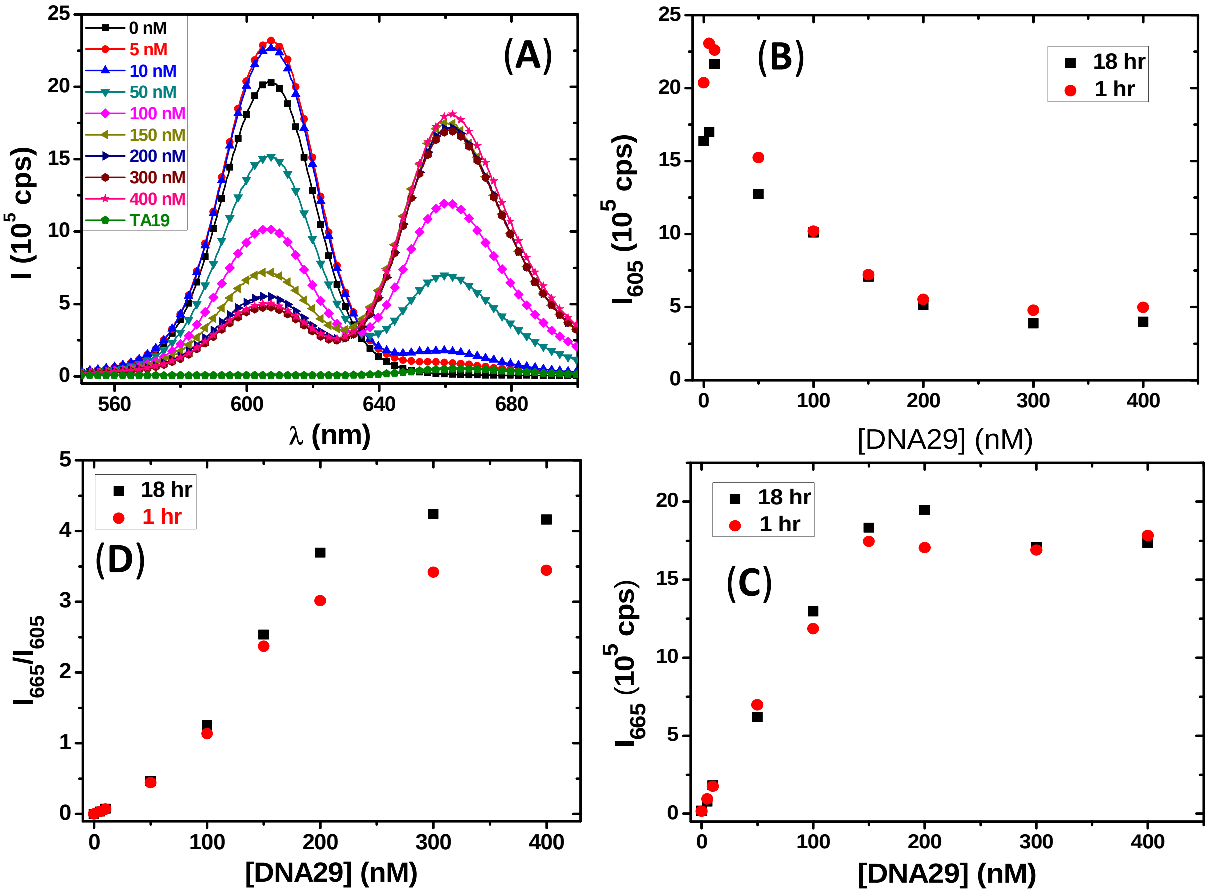

Fig. S4. Stability of the QD-TBA$_{20}$ after hybridization with DNA29 in PBS (with 7 μM peptide/BSA). (**A**) Typical fluorescence spectra of QD-TBA$_{20}$ (10 nM) after hybridization with different amount of DNA29 for 1 hr. The corresponding fluorescence intensity of the QD at 605 nm, I_{605} (**B**), Atto647N FRET signal at 665 nm, I_{665} (**C**), and the I_{665}/I_{605} ratio (**D**) of the system after hybridization for 1h (**filled red circles**) and 18 h (**black squares**), respectively. The I_{605} was found to decrease slightly while the I_{665} FRET signal increased slightly, leading to an increased I_{665}/I_{605} ratio as the hybridization time was increased from 1 to 18 hr. The increase of the I_{665}/I_{605} ratio was minimum at low DNA: QD ratio (≤ 15), but become more apparent as the DNA29:QD ratio was increased to > 20:1, suggesting that it may take some time for the DNA29 to fully hybridize to all of the available the QD conjugated TBA strands at high concentrations or the QD-TBA$_{20}$/DNA29 undergo a slight structural re-arrangement after full hybridization. **Nevertheless, the results presented here clearly show that the hybridized QD-TBA$_{20}$/DNA29 system is highly stable in PBS buffer, a significant improvement over previously reported small-molecule ligand capped QD-DNA sensor systems.**

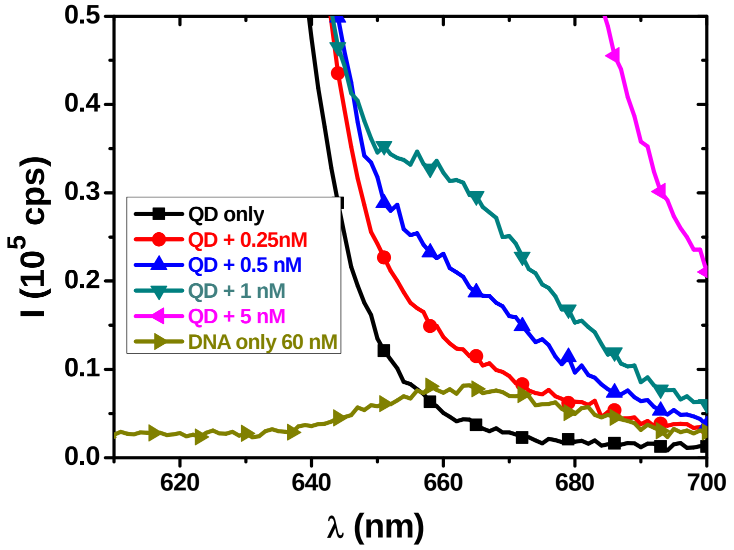

Fig. S5. Amplified fluorescence spectra of the QD-TBA_{20} system (C_{QD} = 2 nM) over the Atto647N fluorescence region (635-700 nm) showing the detection of DNA29 in the sub- to low nM range. It is apparent that the QD sensitized Atto647N FRET signal at 0.25 nM DNA29 is at least as strong as that of 60 nM DNA29 under direct excitation of 450 nm (the λ_{Abs} minimum of Atto647N), suggesting a FRET-to-direct excitation efficiency ratio of this QD-FRET sensor here is at least 240.

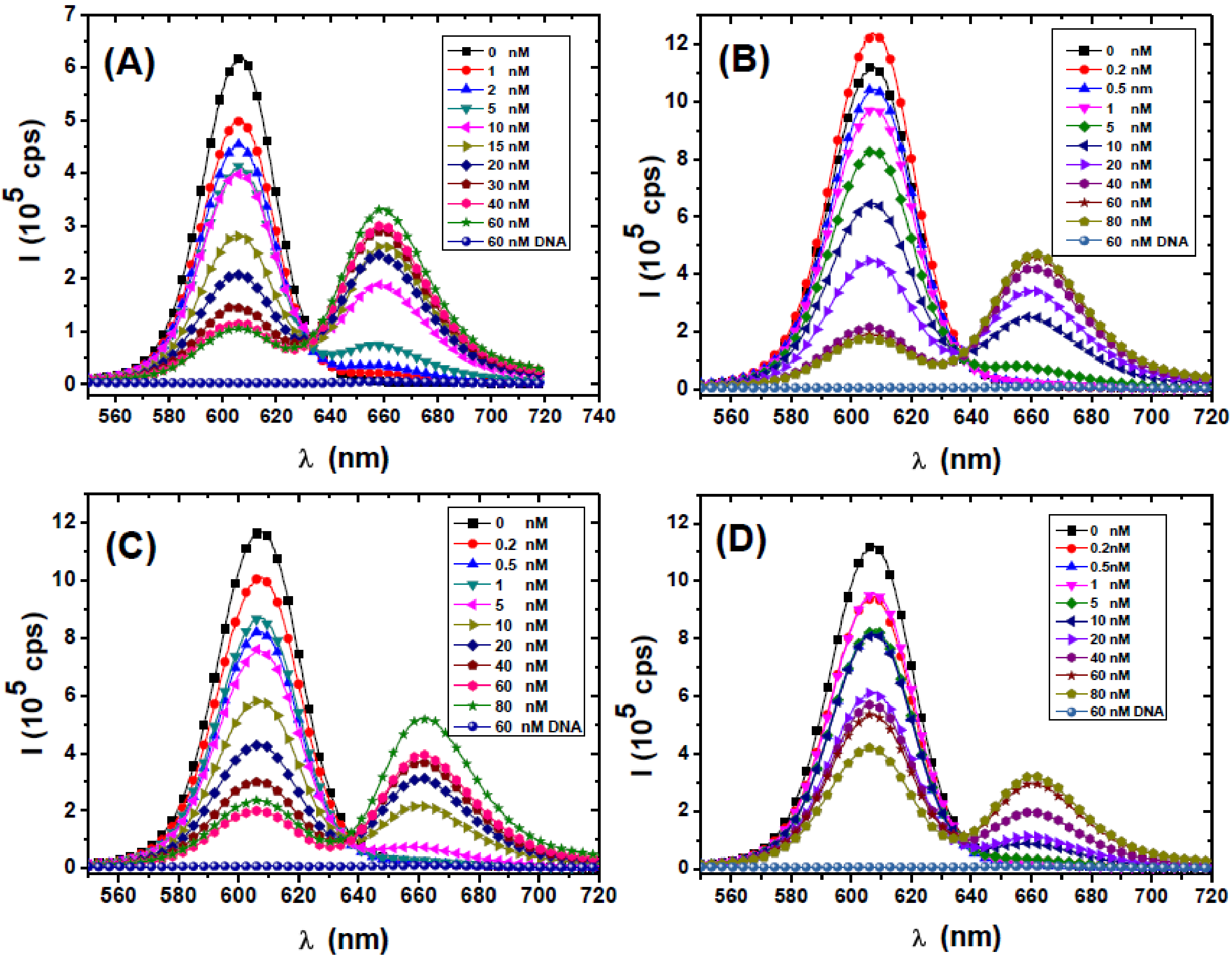

Fig. S6. Representative fluorescence spectra of the QD-TBA_{20} (2 nM in PBS with 7 μM Peptide/BSA) after hybridization to complementary DNA probes with different lengths for 2 hrs at different concentrations. The DNAs used here are: (**A**) DNA18; (**B**) DNA15, (**C**) DNA12 and (**D**) DNA12-SM. A general trend here is that the QD fluorescence peaking at ~650 nm is quenched while the Atto647N FRET signal at ~665 nm is increased with the increasing DNA probe concentration, although the efficiency of the QD fluorescence quenching and QD-sensitised FRET enhancement are strongly dependent on the DNA length and complementary sequence.

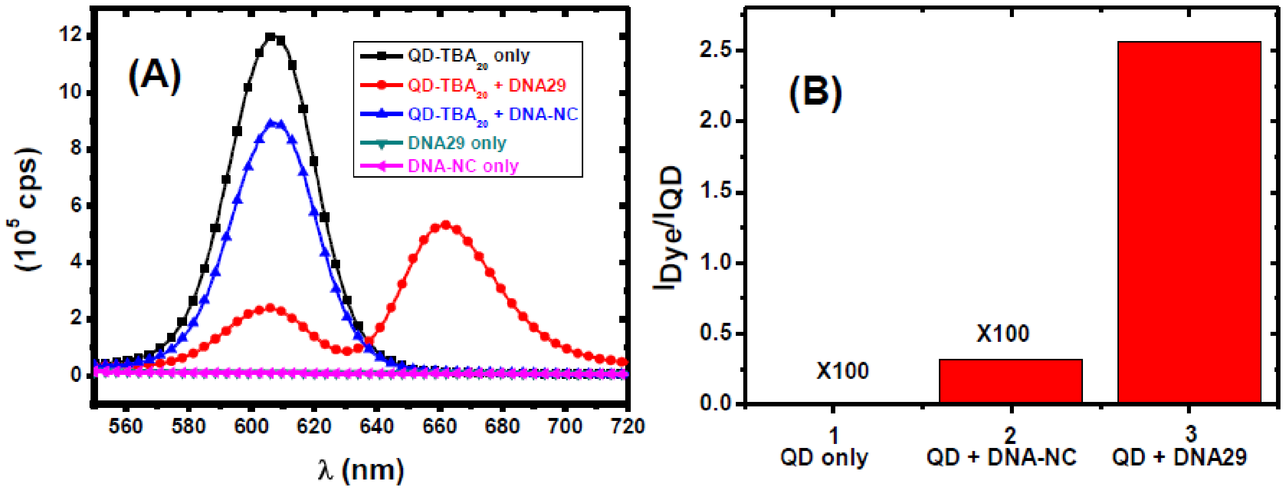

Fig. S7. Specificity of the QD-TBA$_{20}$ based sensor in detecting complementary DNA probes in large excess of bovine serum albumin (BSA, 10 μM) in PBS with 7 μM peptide.

(**A**) Fluorescence spectra of QD-TBA$_{20}$ only (**black line**); QD-TBA$_{20}$ + 30 nM DNA29 (**red**); QD-TBA$_{20}$ + 30 nM DNA-NC (**blue**); 30 nM DNA29 only (**green**); and 30 nM DNA-NC only (**pink**).

(**B**) The corresponding integrated **I_{Dye}/I_{QD}** ratios for QD-TBA$_{20}$ only (**1, QD only**); QD-TBA$_{20}$ + 30 nM DNA-NC (**2, QD + DNA-NC**) and QD-TBA$_{20}$ + 30 nM DNA29 (**3, QD + DNA29**). The I_{Dye}/I_{QD} values for the QD only and (QD + DNA-NC) samples have been amplified by a factor of 100. The **I_{Dye}/I_{QD}** value for DNA29 is **816** times that of the DNA-NC before background (direct excitation Atto647N) correction.

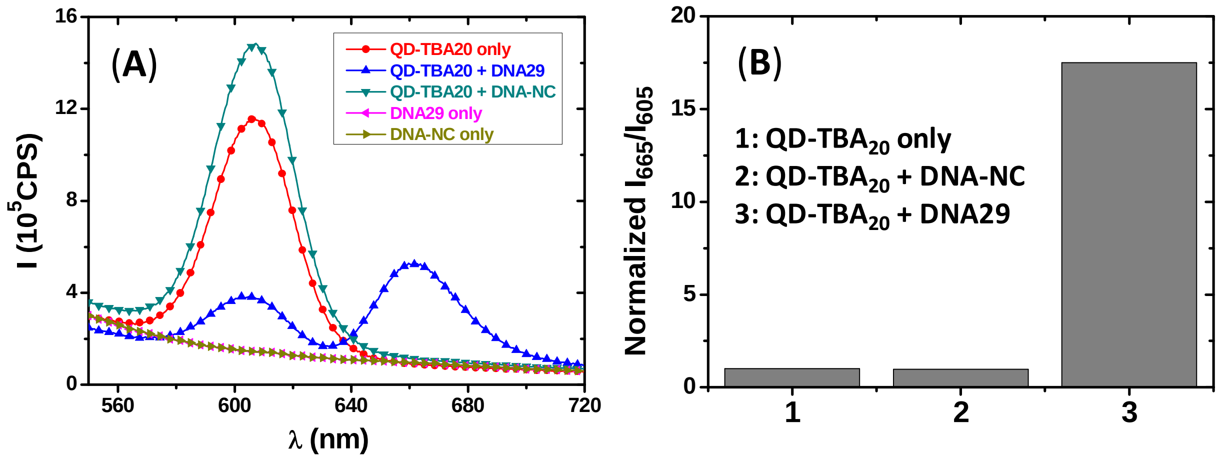

Fig. S8. Specificity of the CFCC clicked QD-TBA$_{20}$ sensor in detecting DNA probes in 10% human serum.

(**A**) Fluorescence spectra of QD-TBA$_{20}$ only (**red**); QD-TBA$_{20}$ + 30 nM DNA29 (**blue**); QD-TBA$_{20}$ + 30 nM DNA-NC (**green**); 30 nM DNA29 only (**purple**); and 30 nM DNA-NC only (**yellow-brown**).

(**B**) The corresponding normalised I_{665}/I_{605} ratios for QD-TBA$_{20}$ only (**1**); QD-TBA$_{20}$ + 30 nM DNA-NC (**2**) and QD-TBA$_{20}$ + 30 nM DNA29 (**3**). The I_{665}/I_{605} ratio for DNA29 is **18** times that of the DNA-NC before correction of the serum background, where the later is essentially identical to the QD-TBA$_{20}$ only sample.

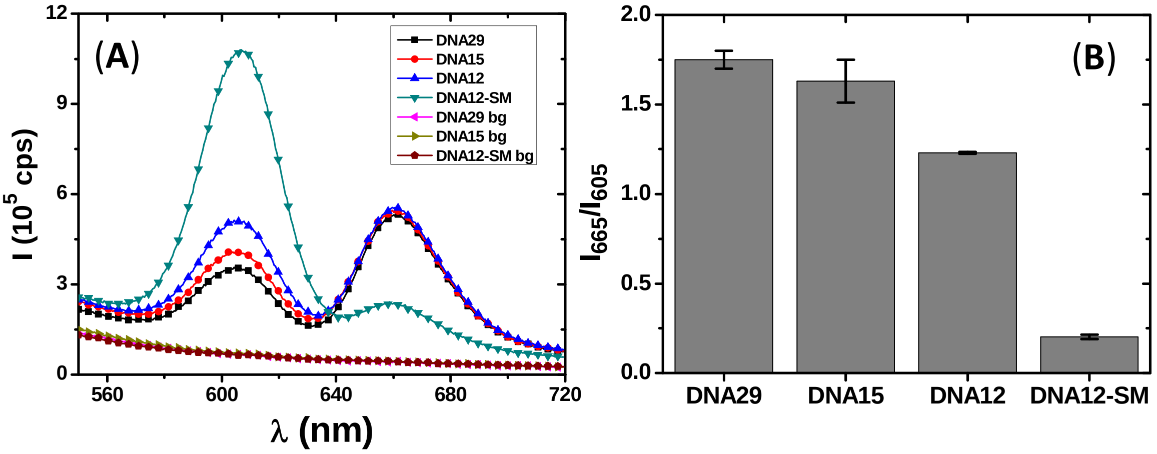

Fig. S9. Discrimination of different length complementary DNA targets in 10% human serum using the CFCC clicked QD-TBA$_{20}$ conjugate. (**A**) Fluorescence spectra of the QD-TBA$_{20}$ (2 nM) after hybridization with 30 nM of different length complementary DNA targets (DNA29, DNA15, DNA12 and DNA12-SM) in 10% human serum. (**B**) The corresponding background corrected I_{665}/I_{605} ratios for the above different DNA targets. The I_{665}/I_{605} values for the DNA29, DNA15 and DNA12 are 8.7, 8.1 and 6.1 times that of DNA12-SM, respectively.

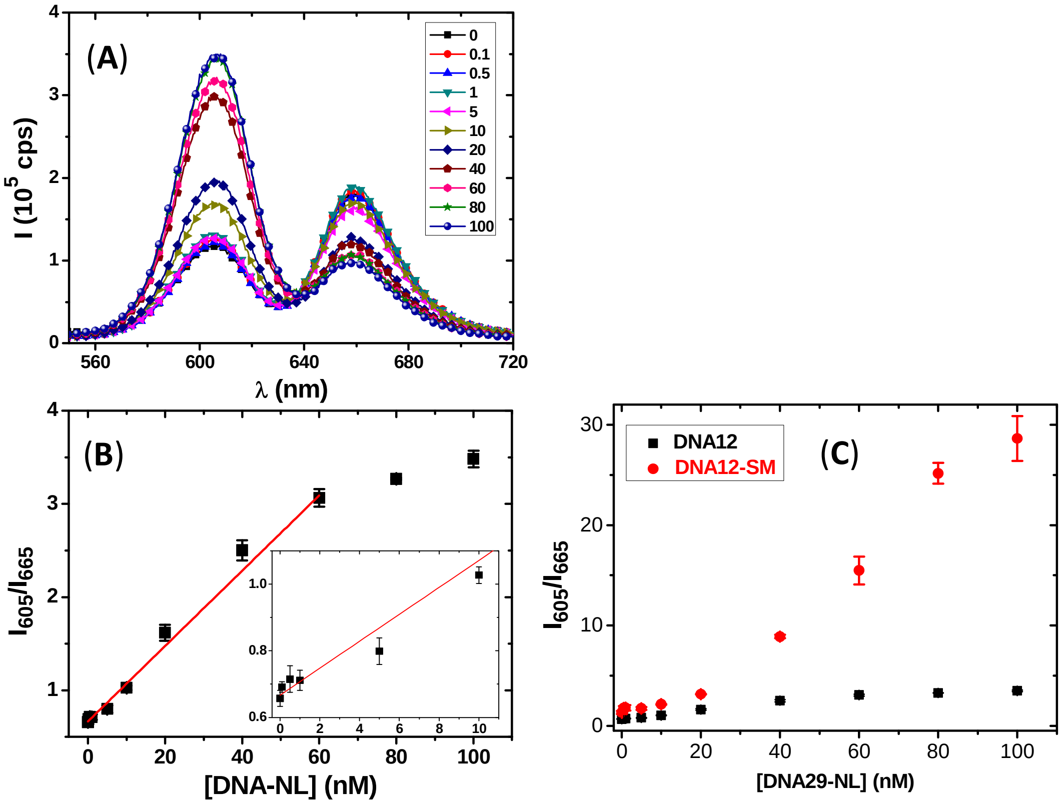

Fig. S10. Label-free detection of the DNA-NF using the CFCC clicked QD-TBA$_{20}$ *via* the DNA strand displacement assay. (**A**) Fluorescence spectra of QD-TBA$_{20}$ pre-hybridized to DNA12 after treatment with different concentrations of DNA29-NL; (**B**) the corresponding I_{605}/I_{665} ratio as a function of [DNA-NL]; and (**C**) comparison of the I_{605}/I_{665} ratio as a function of [DNA29-NL] for QD-TBA$_{20}$ pre-hybridized to DNA12 and DNA12-SM. The QD-TBA$_{20}$ pre-hybridized to DNA12-SM gave a much greater change of the I_{605}/I_{665} ratio as DNA29-NL was added, suggesting more efficient displacement by DNA29-NL.

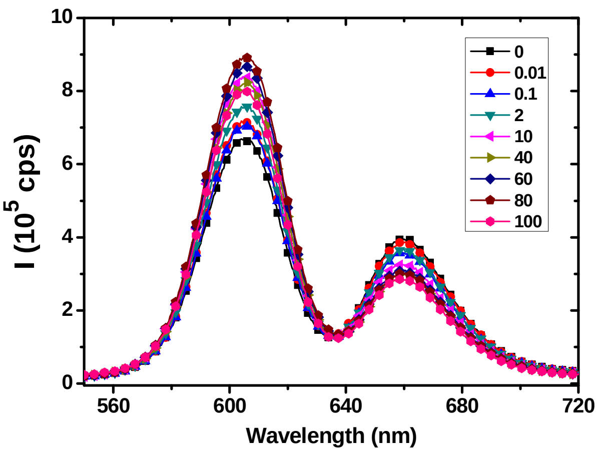

Fig. S11. Typical fluorescence spectra of QD-TBA_{20} (2 nM) stabilized in BSA (10 μM) pre-hybridized with DNA12-SM (80 nM) in PBS for 2 hrs, and then added in different [TB] and was slowed to stand at room temperature for 2 hrs.

Table S1. Summary of the sensing performance of some QD-DNA aptamer sensors for protein detection using fluorescence spectroscopy without target amplifcation.

Sensing Mechanism	Target	LOD (nM) limit	Detectable range (nM)	**Reference**
QD-FRET	Thrombin	10	10-1000	[1]
QD-MB FRET	Thrombin	1	1-500	[2]
ET quenching	Thrombin	10	10-210	[3]
QD-FRET	Mucin-1	250	250-2000	[4]
Electrochemical	Thrombin	~ 1 nM	1-1000	[5]
QD-FRET	Thrombin	0.5	0.01-100	This work

Reference:

[1] Zhang, H.Y., Stockley, P.G. and Zhou, D.J. *Faraday Discuss.* **2011**, *149*, 319-332.
[2] Chi, C. W., Lao, Y. H., Li, Y. S. and Chen, L. C. *Biosens. Bioelectron*. 2011, *26*, 3346-3352.
[3] Raichlin, S., Sharon, E., Freeman, R., Tzfati, Y. and Willner, I. *Biosens. Bioelectron*. **2011**, *26*, 4681-4689.
[4] Cheng, A. K. H., Su, H., Wang, Y. A. and Yu, H.-Z. *Anal. Chem.* 2009, **81**, 6130-6139.
[5] R. Polsky, R. Gill, L. Kaganovsky and I. Willner, *Anal. Chem*. 2006, **78**, 2268.
